# Health benefits of late-onset metformin treatment every other week in mice

**DOI:** 10.1038/s41514-017-0018-7

**Published:** 2017-11-20

**Authors:** Irene Alfaras, Sarah J. Mitchell, Hector Mora, Darisbeth Rosario Lugo, Alessandra Warren, Ignacio Navas-Enamorado, Vickie Hoffmann, Christopher Hine, James R. Mitchell, David G. Le Couteur, Victoria C. Cogger, Michel Bernier, Rafael de Cabo

**Affiliations:** 1 Experimental Gerontology Section, Translational Gerontology Branch, Intramural Research Program, National Institute on Aging, National Institutes of Health, 251 Bayview Boulevard, Baltimore, MD 21224 USA; 2 Centre for Education and Research on Ageing, ANZAC Research Institute, Sydney, NSW Australia; 3 Diagnostic & Research Services Branch, Division of Veterinary Resources, Office of Research Services, National Institutes of Health, Bethesda, MD 20892-2324 USA; 4 Department of Genetics and Complex Diseases, Harvard University, Boston, MA 02115 USA; 5 Sydney Medical School, University of Sydney, Sydney, NSW Australia

## Abstract

Chronic 1% metformin treatment is nephrotoxic in mice, but this dose may nonetheless confer health benefits if given intermittently rather than continuously. Here, we examined the effects of 1% metformin given every-other week (EOW) or two consecutive weeks per month (2WM) on survival of 2-year-old male mice fed standard chow. EOW and 2WM mice had comparable life span compared with control mice. A significant reduction in body weight within the first few weeks of metformin treatment was observed without impact on food consumption and energy expenditure. Moreover, there were differences in the action of metformin on metabolic markers between the EOW and 2WM groups, with EOW metformin conferring greater benefits. Age-associated kidney lesions became more pronounced with metformin, although without pathological consequences. In the liver, metformin treatment led to an overall reduction in steatosis and was accompanied by distinct transcriptomic and metabolomic signatures in response to EOW versus 2WM regimens. Thus, the absence of adverse outcomes associated with chronic, intermittent use of 1% metformin in old mice has clinical translatability into the biology of aging in humans.

## Intermittent use of metformin improves healthspan in old mice

Chronic daily exposure to a high dose of metformin (e.g., 1% w/w) shortens lifespan of non-diabetic mice, although in the short term this treatment confers a similar pattern of gene expression and phenotypes consistent with the benefits of caloric restriction. A team of researchers led by Rafael de Cabo at the National Institute on Aging, NIH tested whether a strategy of intermittent 1% metformin treatment in old mice alters the course of aging and avoids toxicity. They found that when metformin was given every-other-week, it significantly improved insulin sensitivity and reduced age-associated liver lesions without having a negative impact on maximum lifespan in male mice. The absence of adverse outcomes associated with the use of 1% metformin in old mice has clinical translatability into the biology of aging in humans.

## Introduction

Metformin is a biguanide used extensively since the 1960s in the treatment of type 2 diabetes. Metformin has an established safety record and its current approved indication is to improve glycemic control in adults and children with type 2 diabetes mellitus along with diet and exercise.^1^ Recent studies in preclinical models also support a novel role of metformin in improving healthspan and lifespan.^2–6^

The primary target of metformin appears to be the mitochondrion through inhibition of complex I of the respiratory chain^7,8^ and mitochondrial glycerophosphate dehydrogenase (GPD2).^9^ The ensuing perturbations in cellular energy status and redox homeostasis result in activation of the energy sensor AMP-activated protein kinase (AMPK). AMPK-independent actions of metformin have been also described,^10–14^ which include the reduction in mTOR/p70S6K activity resulting from Rag GTPase inhibition.^10^ The anti-gluconeogenic action of metformin is preserved in liver-specific AMPK knockout mice.^13^ Moreover, this biguanide confers protection against certain cancers by inhibiting mTOR/p70S6K activity in an AMPK-independent manner,^15^ and reduces the tumorigenicity of induced pluripotent stem cells while maintaining their pluripotency.^16^ Impaired cellular senescence and accumulation of DNA damage contribute to aging^17^ and metformin has been shown to trigger an immune-mediated clearance of senescent cells by activating an ATM-dependent DNA damage response.^18^ The ability of metformin to lower the incidence of diabetes-associated cardiovascular events and risk of cancer without interfering with stem cell pluripotency may have important implications for a number of age-related diseases that affect lifespan (for recent review^19^).

Metformin is a calorie restriction (CR) mimetic, with several of its metabolic actions, particularly those related to glucose metabolism, resembling to some extent those of CR.^2,3,20,21^ Our previous study showed that diet supplementation with a low dose of metformin (0.1% w/w) improves healthspan and extends lifespan when treatment was started in mice at middle age.^2^ However, long-term exposure to a high dose of metformin (e.g., 1%) is nephrotoxic and leads to early mortality in mice even though global gene expression profile appears to be closer to CR when given only for a short period of time.^2^ Nevertheless, there has been a recent flurry of interest in the use of metformin in clinical translational research on aging. In that regard, a new clinical trial has been set up to investigate the health benefits of chronic metformin treatment and determine whether it can induce dietary restriction-like state in humans (ClinicalTrials.gov Identifier: NCT02745886). A second clinical study, which is known as the “Targeting Aging with Metformin” study, aims at assessing the potential of a pharmacological intervention in delaying age-associated diseases beyond metformin’s isolated impact on diabetes.^22^ A similar paradigm is currently being tested in the pilot study known as “Metformin in Longevity Study” (ClinicalTrials.gov Identifier: NCT02432287). In the latter trial, investigators will search for gene expression changes in adults 60 years and older to provide initial clues on how and whether an older molecular and genomic signature can be reverted to a younger one. These studies will take years before providing meaningful information on the healthspan and lifespan potential of metformin in humans.

In order to provide timely insights into the potential pro-longevity benefits of this drug, we set out to examine the effect of intermittent treatment with 1% metformin given either every-other-week (EOW) or two consecutive weeks per month (2WM) in 2-year-old male mice. We chose EOW and 2WM in order to potentially reduce the impact and incidence of nephrotoxicity observed in our previous study, while conferring CR-like health benefits.^2^ The results indicate that intermittent metformin treatment did not lead to early mortality and that EOW metformin improves some metabolic markers of health without promoting lifespan extension when treatment is initiated in late-life in mice. The mechanisms potentially underlying these effects are discussed.

## Results

### Animal characteristics in response to intermittent metformin treatment

Daily exposure to low dose of metformin (0.1% w/w in diet) confers healthspan benefits and extends lifespan when given to middle-age male mice while a higher dose (1% w/w) was found to be toxic.^2^ Here, we asked whether intermittent feeding with 1% metformin in the diet could confer protection in the absence of toxicity in a cohort of 2-year-old male mice.

**Fig. 1:**
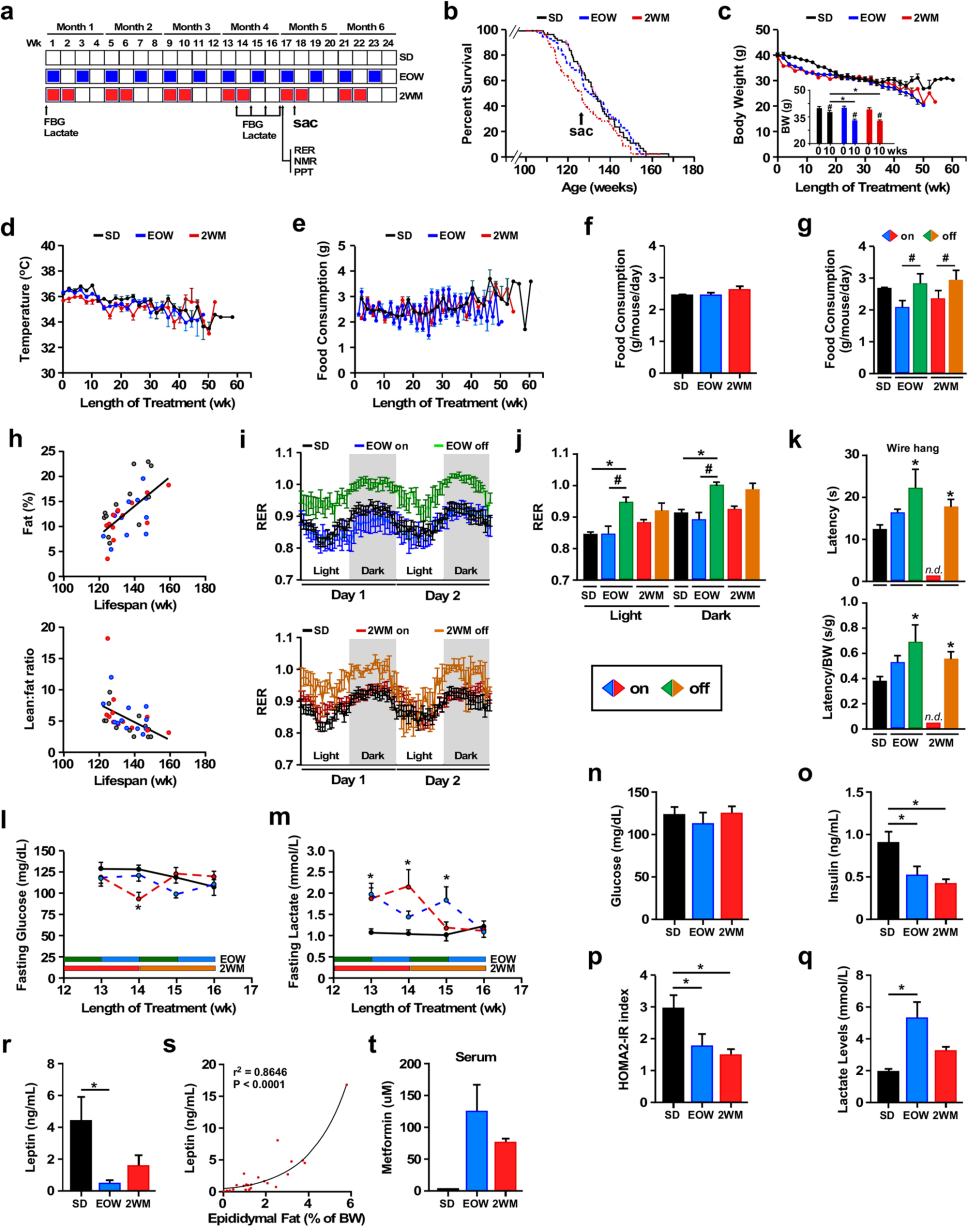
Characteristics of mice on intermittent metformin treatment. **a** Protocol design. Male mice (108 week-old) were fed a standard diet (SD) without (*n* = 68) or with 1% metformin in the diet every-other week (EOW, *n* = 64) or for two consecutive weeks each month (2WM, *n* = 67) for 24 weeks. At the indicated time points (weeks), body composition (NMR), metabolic assessment (RER), and physical performance tests (PPT) were performed on non-fasted animals, whereas blood glucose and lactate levels were measured in 6-h fasted mice. Sac, sacrifice of a subset of animals for tissue collection and analysis (SD, *n* = 10; EOW, *n* = 6; 2WM, *n* = 6). **b** Kaplan–Meier survival curve for the three experimental groups of mice (SD, EOW, 2WM). No extension of maximal lifespan was observed. **c** Body weight profile over the lifespan. Data include all live animals at each time point. *Inset*, Body weight at the initiation (0) and after 10 weeks of treatment (10) (SD, *n* = 68(0) and 64(10); EOW, *n* = 64(0) and 58(10); 2WM, *n* = 67(0) and 57(10)). #*p* < 0.05 compared to *t* = 0; *p < 0.05 compared to SD at *t* = 10 weeks. **d** Body temperature. **e** Food consumption. **f** Average daily food consumption per mouse. **g** The data shown in panel **f** was segregated by whether mice were on metformin treatment or returned to SD without metformin. **h** Correlations between % body fat (upper panel) or lean-to-fat ratio (lower panel) and time of death (*n* = 12 in each group) (upper, *F* = 22.27; dFn, dFd (1, 35); *P* < 0.0001); lower, (*F* = 11.1; dFn, dFd (1, 35); *P* < 0.002). **i**–**j** Mice were subjected to metabolic assessment after 17 weeks of treatment (SD, *n* = 11; EOW on metformin, *n* = 6; EOW off metformin, *n* = 6; 2WM on metformin, *n* = 9; 2WM off metformin, *n* = 3): **i** Respiratory exchange ratio (RER) values over the course of 48 h; **j** RER values shown in panel **i** were segregated based on the light and dark periods of the L12:D12 cycle; **k** Latency to fall from wire hang was measured after 16 weeks of treatment (SD, *n* = 47; EOW on metformin, *n* = 12; EOW off metformin, *n* = 7; 2WM on metformin, *n* = 0; 2WM off metformin, *n* = 22) before (upper panel) and after (lower panel) correction for body weight. **l**,** m** At 13–16 weeks of treatment, mice were fasted for 6 h (SD, *n* = 8–10; EOW, *n* = 9–10; 2WM, *n* = 9–10) and circulating levels of **l** glucose and **m** lactate were measured. **n–r** The following analyses were carried out at 17 weeks of treatment in fed mice (SD, *n* = 10; EOW, *n* = 6; 2WM, *n* = 6): **n** blood glucose; **o** serum insulin levels; **p** HOMA-IR index; **q** blood lactate; **r** Serum levels of leptin. **s** Correlation between circulating levels of leptin and amount of epididymal fat as percent body weight. **t** Serum levels of metformin after 17 weeks of treatment (SD, *n* = 3; EOW, *n* = 6; 2WM, *n* = 6). Data are represented as the mean ± s.e.m. *P < 0.05 compared to SD-fed mice, #*P* < 0.05 compared to mice on metformin (Kruskal–Wallis with Dunn’s post hoc test)

Male C57BL/6 mice were fed a standard AIN-93G diet (SD) supplemented with 1% metformin EOW or for 2 consecutive weeks each month (2WM) for the remainder of their lives (*n* = ~65 mice per experimental group × 3 groups). The collection of blood samples and measures of physiological/biochemical parameters was carried out as depicted in Fig. 1a. EOW and 2WM metformin treatment did not alter mean or maximum lifespan of mice (Fig. 1b, Supplementary Table S1), and gross necropsy examination on all mice that died revealed no histopathological differences between experimental groups (Supplementary Table S2) consistent with the lifespan data. The absence of major renal pathologies at the time of death in mice subjected to intermittent 1% metformin treatment was in sharp contrast to the apparent renal failure observed in mice on chronic, daily 1% metformin.^2^ EOW and 2WM mice consumed about 1 g metformin kg^−1^ body weight per day, which translates to about 80 mg metformin kg^−1^ body weight in humans,^23^ a dosage that is more than twice the maximum dose of metformin that patients receive (3 × 850 mg per day). Weight trajectory of the three groups is shown in Fig. 1c, with a greater decrease of weight in the first few weeks of treatment in EOW and 2WM mice compared to control animals. While the SD group lost 2.04 g by 10 weeks, metformin treatment caused a significant weight reduction by more than 6.98 and 6.51 g in EOW and 2WM mice, respectively (Fig. 1c inset). All three groups demonstrated comparable body weight trajectories by 20 weeks of treatment. The body temperature and food consumption levels were also similar over the ~55-week treatment (Fig. 1d, e). Although the average daily food consumption per mouse was unaffected (Fig. 1f), there was significant reduction in food intake when mice were on metformin compared to mice getting off the drug (Fig. 1g). Intermittent metformin treatment maintained the positive association between % body fat (measured by nuclear magnetic resonance (NMR) after 16 weeks of treatment) and lifespan (Fig. 1h, upper panel), which coincided with an inverse relationship between the lean-to-fat ratio and maximal survival (Fig. 1h, lower panel).

To assess the effect of metformin on in vivo energy metabolism, indirect calorimetry was performed for 48 h using the CLAMS system. The daily cycling of the respiratory exchange ratio (RER; CO_2_ production vs O_2_ consumption) in old EOW and 2WM mice while on metformin was similar to that of SD mice (Fig. 1i–j). However, a significant increase in RER values in both the light and dark cycles was observed upon the return of EOW mice to SD without metformin, with a similar trend in 2WM mice, indicating a proportional substrate preference toward carbohydrate utilization for energy demands once metformin was removed from the diet (Fig. 1i–j). Under these conditions, locomotor activity was undistinguishable between experimental groups (Supplementary Fig. S1a, S1b). The experiments suggest that the intermittent removal of metformin may have influenced RER in aged animals.

A number of behavioral and locomotor tests was then performed when old mice were on metformin and after they were returned to SD without metformin. Overall, the removal of metformin was associated with significant increase in the latency to fall from a wire hang test in both groups of mice (Fig. 1k), but not after using the elevated wire cage top test or an accelerating rotarod (Supplementary Fig. S1c), indicating a rather mild improvement, if any, in motor coordination and learning. The grip strength of mice was also unresponsive to metformin treatment with or without correction for body weight (Supplementary Fig. S1c).

We undertook a study where serum samples were collected from four consecutive weeks (week 13–16) to determine circulating glucose and lactate levels after a 6-h fast. While being unaffected in EOW mice, the fasting blood glucose levels were significantly lower in 2WM mice at week-14, representing the second consecutive week on metformin (Fig. 1l). Increase in plasma lactate levels is common in response to metformin due to inhibition of hepatic mitochondrial respiration.^24^ Here, fasting plasma lactate concentrations significantly increased when mice were exposed to metformin before returning to basal levels during the off-periods (Fig. 1m). An additional blood chemistry analysis was performed in non-fasting animals at 17 weeks of treatment when both EOW and 2WM mice were on metformin. There was significant improvement in insulin levels and homeostatic model assessment of insulin resistance (HOMA-IR) that did not translate in lower blood glucose when compared to SD controls (Fig. 1o–p versus Fig. 1n). Glycated hemoglobin A1c (HbA1c, an indicator of the 3-mo average plasma glucose concentration) and serum adiponectin levels were comparable in all groups (Supplementary Fig. S1d). EOW mice exhibited significant increase in circulating lactate (Fig. 1q) that coincided with a marked reduction in leptin levels (Fig. 1r) despite similar lean-to-fat ratio as SD mice (Supplementary Fig. S1e). Comparable trend was observed in metformin-treated 2WM mice. As anticipated, a positive and significant correlation between epidydimal fat content and serum leptin levels was observed (Fig. 1s). Although no further tests to evaluate glucose or insulin tolerance were conducted, we observed that the triglyceride levels in EOW livers trended lower from 54.97 ± 15.68 (SD) to 24.46 ± 4.36 mg g^−1^ (Supplementary Fig. S1f), consistent with a possible improvement in insulin sensitivity.^25^ Hydrogen sulfide production mediates multiple benefits normally associated with stress resistance and longevity.^26^ Here, there was a near significant increase in hepatic production of hydrogen sulfide in EOW mice, compared to SD livers (*P* = 0.051; Supplementary Fig. S1g). Metformin was detected in the serum and liver extracts of EOW and 2WM mice after 17 weeks of treatment (Fig. 1t and Supplementary Fig. S1h). These results indicate a slight improvement in the metabolic health of the aged mice exposed to intermittent metformin.

### Effect of intermittent metformin treatment on liver and kidney histology

**Fig. 2:**
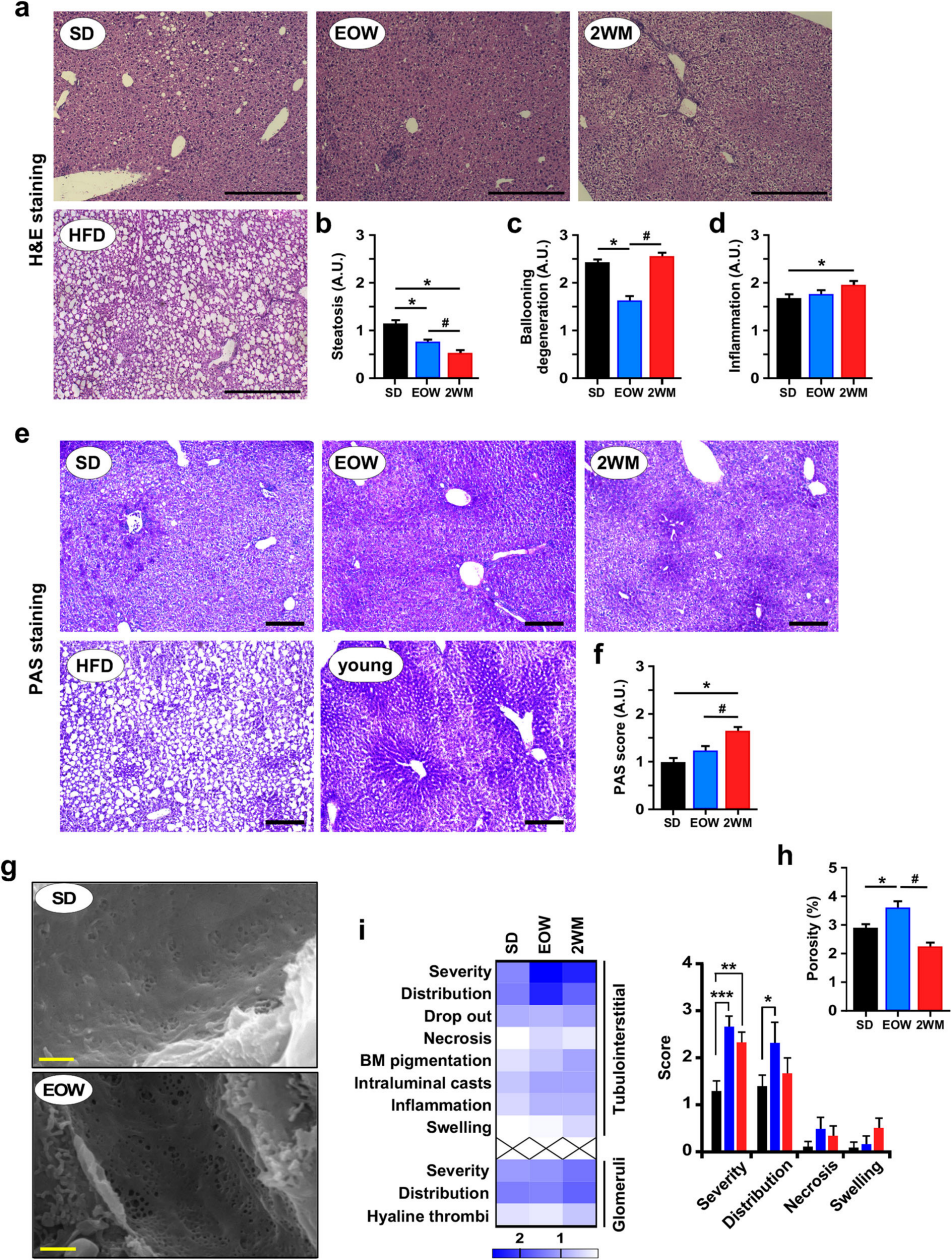
Intermittent metformin treatment reduces liver injury and improves the porosity of the mouse liver sieve. **a** H&E staining depicted steatosis as circular white gaps caused when the dehydration process leaches the fat out of fixed liver tissues. HFD, representative image of fixed liver section of a high-fat diet-fed mouse showing intense steatosis. **c-d** The following quantitative measurements were carried out: **b** The degree of steatosis; **c** Extent of ballooning degeneration of hepatocytes; **d** Degree of inflammation. **e** Periodic acid–Schiff staining (PAS) for the detection of polysaccharides (e.g., glycogen) in fixed liver tissues. Young crtl, representative image of fixed liver section of a young mouse with strong glycogen deposition. **f** Semi-quantification of PAS staining. **a** Scale bar, 200 μm; 5× final magnification; **e** Scale bar, 200 μm; 10× final magnification. **g** Representative scanning electron microscopy images of liver sections of the SD and EOW groups of mice. Scale bar, 1 μm; 15,000× final magnification. **h** The degree of porosity seen as the fenestration area expressed as percent of total area was quantitatively determined. All data are represented as the mean ± s.e.m. *P < 0.05 compared to SD-fed mice (ANOVA with Tukey’s multiple comparisons test and Kruskal–Wallis with Dunn’s post hoc test for PAS). #*P* < 0.05 compared to EOW-treated mice (ANOVA with Tukey’s multiple comparisons test). **i** Heatmap of the average score of various types of tubular and glomerular lesions in each experimental group of mice (SD, *n* = 10; EOW, *n* = 6; 2WM, *n* = 6). Bars represent the mean ± s.e.m. of selected tubulointersitial lesions. See “Methods” for additional information. *P < 0.05, **P < 0.01, ***P < 0.001. SD standard diet

Aging is associated with lipid accumulation and local inflammation.^27^ Histochemical analyses were carried out on frozen-fixed liver tissues to determine the effects of intermittent metformin treatment on hepatic steatosis, ballooning, inflammation, and glycogen deposition (Fig. 2a–f). Compared to control mice, H&E staining of EOW and 2WM livers (Fig. 2a) revealed a signature consistent with a reduction in steatosis (Fig. 2b), even though biochemical quantification of hepatic triglycerides showed only a trend toward lower hepatic lipid accumulation (Supplementary Fig. S1e). EOW livers displayed significant reduction in ballooning degeneration of hepatocytes in the absence of inflammation (Fig. 2c, d) or change in glycogen deposition (Fig. 2e, f). However, 2WM treatment resulted in higher inflammation and periodic acid–schiff staining (PAS) depots compared to SD controls, thus highlighting differences in the effect of metformin treatment between the EOW and 2WM livers.

Liver sinusoidal endothelial cells contain transcellular pores known as fenestrations that enable transfer of metabolites between blood and the surrounding hepatocytes.^28^ Scanning electron microscopy (SEM) of fixed liver sections was performed to assess the integrity of the hepatic sinusoidal endothelium in mice subjected to intermittent metformin treatment (Fig. 2g). EOW mice exhibited greater porosity of the liver sieve than the SD and 2WM groups (Fig. 2h), and was consistent with improved insulin sensitivity.

Kidney sections from SD, EOW and 2WM mice after 17 weeks of treatment were examined and scored for possible toxic lesions. Both H&E and PAS-stained slides were evaluated by a certified veterinary pathologist. All kidneys had age-associated background tubular lesions of varying degrees while glomerular lesions were milder, suggesting that injury originated with the tubules (Fig. 2i). Metformin treatment contributed to an increase in renal tubular lesions in the EOW and 2WM cohorts compared to SD controls, which included flattening of renal tubular epithelial cells with loss of brush borders, ceroid pigmentation of basement membranes, and mild interstitial inflammation (Fig. 2i and data not shown). The results indicate that this increased incidence in renal lesions upon EOW and 2WM metformin has little effect, if any, on maximal lifespan.

### Hepatic gene expression profiles in mice under intermittent metformin treatment

**Fig. 3:**
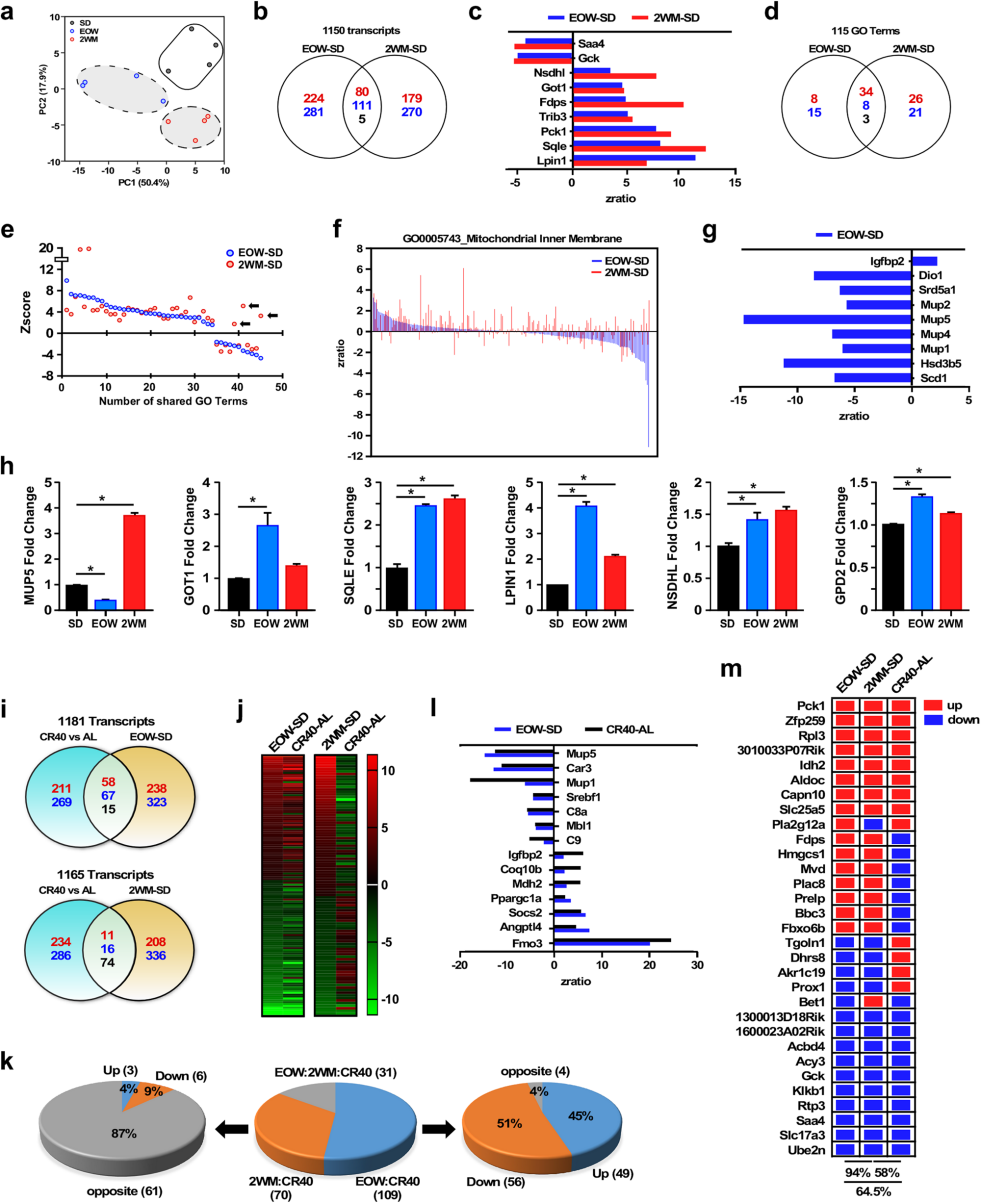
Intermittent metformin treatment alters the global hepatic gene expression profile of SD-fed mice. **a** Principal component analysis (PCA) from microarray RNA experiments in livers of 108-week-old mice maintained for 17 weeks on 1% metformin either EOW or 2WM compared with SD controls (SD, *n* = 4; EOW, *n* = 4; 2WM, *n* = 4). **b** Venn diagram of upregulated (red), downregulated (blue), and reciprocally regulated (black) gene transcripts between the EOW–SD and 2WM–SD pairwise comparisons. **c** Graphical representation of select genes significantly impacted by EOW and 2WM. As indicated in Methods, the measure in gene expression uses *Z*-score transformation of the normalized data, accompanied by *z*-ratios for predicting significant changes.^73, 74^ **d** Venn diagram of upregulated (red), downregulated (blue), and reciprocally regulated (black) GO Terms between the EOW–SD and 2WM–SD pairwise comparisons. **e** Graphical representation of the significant 45 GO Terms shared by EOW (plotted in blue) and 2WM (plotted in red). Zscores depict the number of standard deviations, either above or below the mean of all pathways, the aggregated *Z*-score of a given GO Term (or pathway) has within a pairwise comparison (see Methods for additional details). The list of all the significantly modified GO Terms can be found in Supplementary Table S4c. Arrows depict 3 GO Terms with changes (*Z*-score) shifted in the opposite direction between the two pairwise comparisons. **f** Expression of genes within the GO Term “Mitochondrial inner membrane” is depicted as *Z* ratios. **g** Expression of genes significantly altered only in the EOW–SD comparison. **h** Validation of the microarray data by quantitative RT-PCR. Data are represented as the mean ± s.e.m. *P < 0.05 compared to SD-fed mice (*t*-test two tailed). SD, standard diet. **i–m** Exploratory data analysis aimed at visualizing the main characteristics of the current set of gene expression with that of our recent study on the chronic effect of 40% calorie restriction versus ad libitum feeding (CR40-AL) in male C57BL/6J mice.^32^ **i** Venn diagram of upregulated (red), downregulated (blue), and reciprocally regulated (black) gene transcripts in the livers of CR40-AL versus either EOW–SD (upper panel) or 2WM–SD (lower panel) pairwise comparisons. **j** Heat map comparing the expression of liver transcripts common between EOW–SD and CR40-AL or 2WM–SD and CR40-AL. **k** Distribution of the 210 transcripts shared between EOW–SD and CR40-AL (109), 2WM–SD and CR40-AL (70), or all three comparisons (31) (middle panel). More than 87% of transcripts shared between 2WM–SD and CR40-AL (61/70) were in the opposite direction (left panel), whereas the large majority (96%) of transcripts shared between EOW–SD and CR40-AL followed the same direction (right panel). **l** Change in expression of select genes common between EOW–SD and 40CR-AL is depicted as *z*-ratios. **m** Binary representation of gene expression related to the 31 transcripts (**k**, middle panel) shared between the three pairwise comparisons. Upregulated, red boxes; downregulated, blue boxes. The complete list of genes is provided in the Supplementary Table S5c

Whole-genome microarray analysis was performed on liver samples of 2-year-old mice after 17 weeks on SD supplemented or not with metformin. Principal component analysis (PCA) showed a clear effect of metformin treatment on liver transcriptome (Fig. 3a). To understand the determinants contributing to the metformin response, two-way Venn diagrams were plotted with the EOW–SD and 2WM–SD pairwise comparisons (Fig. 3b). The number of transcripts that were upregulated (red color) and downregulated (blue color) are depicted, with more than 196 transcripts that were shared. Heat map of the top 100 shared genes indicated great similarity in both the magnitude and direction of gene expression (Supplementary Fig. S2a), as only five transcripts were regulated in opposite direction, including *Idh1* and *Sc5d*. A list of the top 50 significantly altered transcripts shared in EOW and 2WM livers can be found in Supplementary Table S3. In order to predict significant changes in gene expression, the *Z*-score transformation method accompanied by *z*-ratios was used (see Supplementary Information for further details). The effects of metformin were accompanied by marked upregulation of *Lpin1*, *Pck1*, and several genes implicated in cholesterol biosynthesis, with the downregulation of glucokinase (*Gck*) and *Saa4* whose transcript encodes the inflammation-inducible serum amyloid A4 protein (Fig. 3c). *Lpin1* encodes for lipin-1 protein, which acts as a nuclear transcriptional coactivator for the PGC1α/PPARα heterocomplex linked to fatty acid β-oxidation.^29^

Parametric analysis of gene set enrichment enabled the identification of significantly altered genes within 115 GO Terms, 23 of which were uniquely present in the EOW–SD comparison (Fig. 3d, Supplementary Table S4a). The top downregulated GO Term in EOW livers was GO0006633 ‘fatty acid biosynthetic process’ with a *Z*-score of −5.604. Positive and negative scores reveal the number of standard deviations the aggregated *Z*-score of a given GO Term (or pathway) within a pairwise comparison (e.g., EOW–SD) is either above or below the mean of all pathways (See Supplementary Information for additional details). Among the top GO Terms that were impacted in the 2WM–SD comparison were GO0008610 “lipid biosynthetic process” and GO0006350 “transcription” with *Z*-scores of +13.913 and −4.459, respectively (Supplementary Table S4b). Forty-five GO Terms were shared between EOW and 2WM livers, which included the upregulation of pathways implicated in ribosome/translation, biosynthetic and metabolic processes, and signal transduction as well as the downregulation of GO Terms associated with histone acetylation and nucleosome assembly (Supplementary Table S4c). Among the shared GO Terms, only three exhibited an opposite direction in expression, which included GO0005743 ‘Mitochondrial Inner Membrane’ and GO0005739 ‘Mitochondrion’ (Fig. 3e, arrows). The gene expression pattern within the shared GO terms ‘cholesterol biosynthetic process’ and ‘mitochondrial inner membrane’ is depicted (Supplementary Figs. 2b, 3f).

A deeper look into the liver microarray data revealed a number of genes implicated in steatosis and insulin sensitivity that were differentially regulated by intermittent metformin treatment. For instance, expression of *Scd1* and *Hsd3b5*, which strongly correlates with the degree of hepatic steatosis^30^ was found to be significantly lower in EOW compared to 2WM and SD control livers (Fig. 3g). Major urinary protein (MUP) 1 regulates energy expenditure by restricting glucose production and lipogenesis though direct inhibition of hepatic gene expression in diet-induced obese mice.^31^ Here, large reduction in *Mup5, Mup4, Mup1, and Mup2* levels was observed in EOW than in 2WM and SD livers (Fig. 3g). The decrease in hepatic *Mup* expression observed with EOW was comparable to our recently published data in male mice maintained on 40% CR.^32^ The hepatic production of MUPs is increased by a number of hormones, including testosterone, thyroxine, and growth hormone (GH).^33^ As shown in Fig. 3g, EOW livers exhibited significant reduction in expression of *Srd5a1*, a gene implicated in the conversion of testosterone into the more potent androgen, dihydrotestosterone, and *Dio1*, which converts thyroxine to the bioactive thyroid hormone, whereas both genes were unaffected in 2WM livers. Both fasting and metformin treatment stimulate hepatic *Igfbp-2* transcription,^34,35^ which is known to negatively regulate GH signaling. The expression of *Igfbp2*, whose transcript participates in the reduction in the bioavailability of IGFs, was upregulated in EOW, but not 2WM livers (Fig. 3g). Thus, EOW supplementation with metformin was associated with possible dampening in hormonal regulation of Mup gene expression. A select group of genes were validated by real-time quantitative PCR (Fig. 3h). Lastly, immunoblot analysis indicated that the steady-state levels of active AMPK phosphorylated at Thr-172, a known endpoint of metformin action in liver, were comparable in the three experimental groups (Supplementary Fig. 2c, d). Similarly, phosphorylation of ACC at Ser-79, a target of AMPK activity, was unresponsive to metformin treatment, even though the drug reached the liver in sizeable concentrations.

To assess whether intermittent metformin treatment exhibits CR-like transcriptomic profiles, we compared the main characteristics of the current set of gene expression with that of our previous study performed in the livers of ~2-year-old male C57BL/6J mice on 40% CR (CR40).^32^ Details about the experimental cohorts and the way the data was analyzed can be found in Supplementary Methods. Pathway analysis showed a CR40-dependent enrichment in gene sets associated with gluconeogenesis and mitochondrial bioenergetics with the downregulation of several gene sets implicated in inflammation when compared to ad libitum (AL)-fed littermates (Supplementary Fig. S2e). Two-way Venn diagrams were then plotted with the CR40-AL and either EOW–SD or 2WM–SD pairwise comparisons (Fig. 3i). In both data sets, the control groups (AL and SD) were comparable. Heat map suggested great similarity in the magnitude and direction of expression of genes shared between CR40 and EOW livers, but marked divergence between CR40 and 2WM livers (Fig. 3j). Notably, more than 210 genes were shared among the three pairwise comparisons, of which 52% (109/210) and 33% (70/210) appeared to be absent in 2WM and EOW, respectively, and only 15% (31/210) were common for the three comparisons (Fig. 3k, middle panel). More than 87% of the genes (61/70) shared between CR40 and 2WM exhibited an opposite direction of expression (Fig. 3k, left panel; Supplementary Table S5a), whereas only 4% of the genes (4/109) common to both CR40 and EOW displayed similar behavior (Fig. 3k, right panel; Supplementary Table S5b). Among the genes common to EOW and CR40 livers whose magnitude and direction of expression were similar included *Mup5*, *Srd5a1*, *Dio1*, and *Igfbp2* as well as select genes such as *Srebf1*, *Ppargc1a*, and *Socs2* (Fig. 3l). Moreover, *Fmo3* was the top upregulated gene in the EOW–SD and CR40-AL pairwise comparisons, with zratios of +20.36 and +24.89, respectively (Supplementary Table S5b, Fig. 3i). *Fmo3* encodes the hepatic drug-metabolizing enzyme, flavin-containing monooxygenase 3, a member of a conserved family of enzymes with health and lifespan-promoting activities.^36^ A significant increase in the expression of *Angptl4*, whose transcript encodes the hepatic Fibrinogen/Angiopoietin-Related protein (ANGPTL4), was also found in EOW and CR livers (EOW–SD, zratio + 7.57; CR40-AL, zratio + 5.56) (Supplementary Table S5b).

A binary representation of the 31 significantly altered genes shared between CR40, EOW, and 2WM indicated a subset of transcripts with opposite direction of expression between intermittent metformin treatment and CR (Fig. 3m, Supplementary Table S5c): These included *Fdps*, *Hmcgs1*, and *Mvd*, which are primarily involved in cholesterol and sterol biosynthesis.

### Intermittent metformin treatment impacts liver and serum metabolomics

**Fig. 4:**
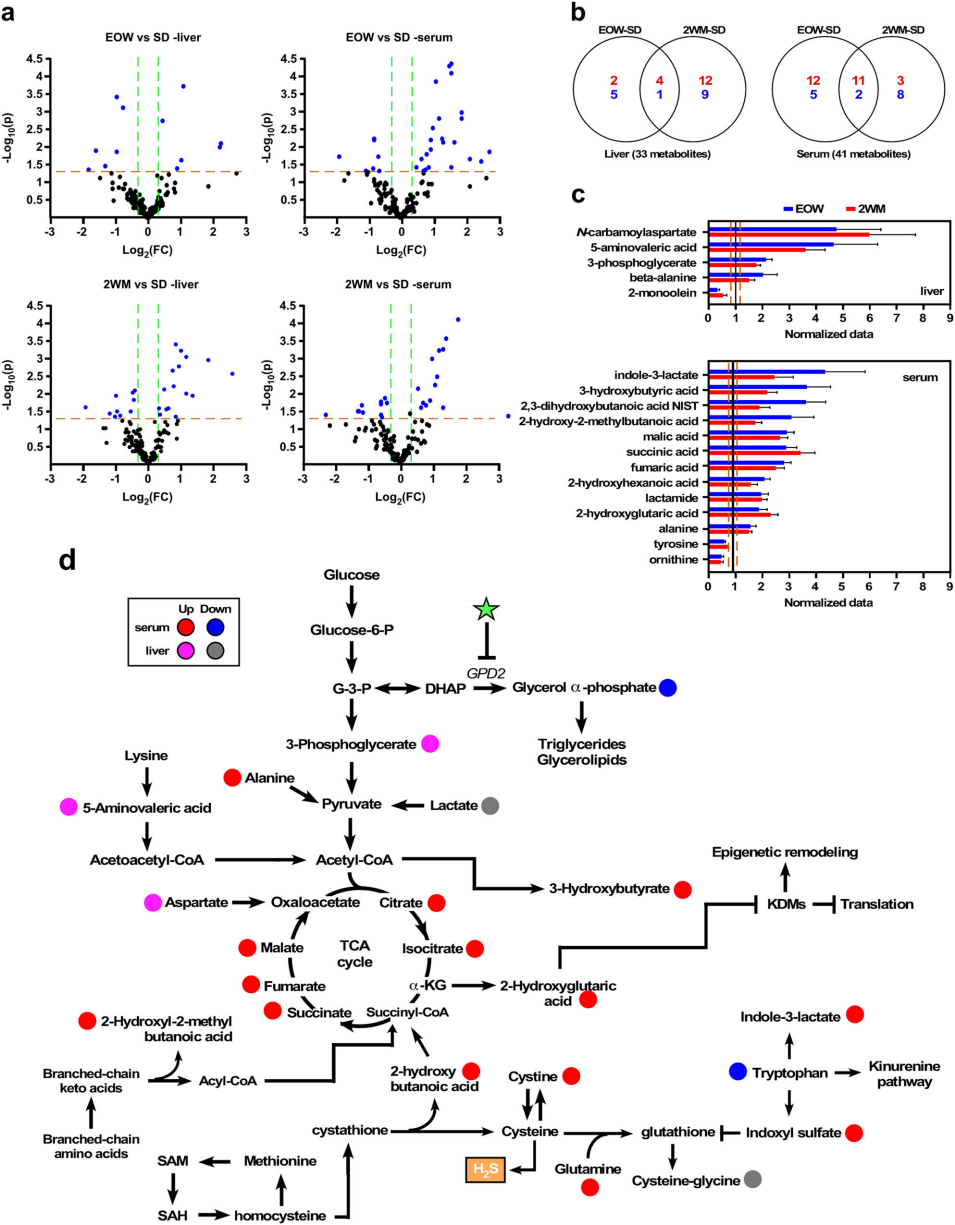
Identification of hepatic and serum metabolites in SD-fed mice subjected to intermittent metformin treatment. **a** Volcano plots of 136 metabolites quantified in the liver and serum of 108-week-old mice maintained for 17 weeks on EOW or 2WM (Liver: SD, *n* = 10; EOW, *n* = 5; 2WM, *n* = 6; serum: SD, *n* = 10; EOW, *n* = 6; 2WM, *n* = 6). Significantly altered metabolites are labeled in blue (fold change ≥ 1.25 in both directions, *P* ≤ 0.05). **b** Venn diagrams of upregulated (red) and downregulated (blue) metabolites in the livers (left panel) and serum (right panel) of mice following EOW–SD and 2WM–SD pairwise comparisons. **c** Graphical representation of the significant metabolites shared by EOW (plotted in blue) and 2WM (plotted in red) normalized to SD controls in the liver (upper) and serum (bottom). See Supplementary Tables S6a and S6b for complete list of significantly altered metabolites. Data are represented as the mean ± s.e.m. **d** Schematic diagram of select metabolic pathways induced by EOW treatment based on the liver and serum metabolite profiles. Metabolism of organic sulfur compounds is also depicted. Red and pink colors represent increased metabolites and blue and gray colors represent decreased metabolites in serum and liver, respectively. H_2_S, production of hydrogen sulfide measured in Supplementary Fig. S1g. Data are represented as the mean ± s.e.m; *n* = 6 biological replicates per group, 23–24 months of age, 17–18 months on diet

More than 150 metabolites resulting from multiple cellular and biological processes were identified in liver and serum samples by untargeted liquid and gas chromatography coupled with mass spectrometry. Volcano plots were used to visualize changes in metabolite levels in response to EOW or 2WM treatment versus SD controls (Fig. 4a). The metabolites significantly impacted by the intermittent metformin treatment (fold change ≥1.25; *P* ≤ 0.05) were merged in two-way Venn diagrams as a mean to identify unique and overlapping ones (Fig. 4b). The magnitude and direction of the levels of metabolites shared between EOW and 2WM were identical in both the serum and liver extracts (Fig. 4c; Supplementary Table S6), and included 3-hydroxybutyric acid, a ketone body, 2-hydroxy-2-methylbutyric acid, whose buildup results from a defect in branched-chain amino acid metabolism, and 2-hydroxyglutarate.

Important metabolites associated with energy and lipid metabolism were found to be specifically reduced in EOW mice (e.g., glycerol α-phosphate and lactate), while other molecules such as aspartate, citrate and isocitrate were increased (*p* < 0.05) (Supplementary Fig. S3a, Fig. 4d). Glycerol α-phosphate is oxidized by GPD2 for transfer of cytosolic NADH to the mitochondrial electron transport chain, and interestingly, the glucose-lowering effect of metformin has been attributed to inhibition of this enzyme.^9^ The drop of glycerol α-phosphate levels in EOW mice correlated with lower hepatic triglyceride levels and decreased susceptibility to age-associated metabolic disorder, e.g., hyperinsulinemia and insulin resistance, and liver steatosis. Moreover, the improvement in systemic insulin sensitivity after intermittent metformin treatment in EOW mice was accompanied by significant reduction in hepatic palmitoleic acid, a monounsaturated fatty acid that is produced almost exclusively through desaturation of palmitic acid by stearoyl-CoA desaturase, whose gene expression level was also reduced specifically in EOW livers (*z* ratio of −6.65; Fig. 3g). The increased levels of 2-hydroxybutanoic acid in serum of EOW mice were consistent with greater cleavage of cystathionine to cysteine, which correlated with accumulation of cystine, the oxidized form of cysteine, and hepatic hydrogen sulfide (Fig. 4d). Lastly, metabolites profiling uncovered the presence of elevated serum levels of the tryptophan metabolites 3-indoxyl sulfate and indole 3-lactate with concomitant depletion in tryptophan in EOW mice (Fig. 4d).

## Discussion

Although the use of metformin as a pro-longevity strategy has been proposed when started early in life,^3^ our analysis reveals that the intermittent use of the drug confers some health benefits in the absence of extended lifespan when the treatment was initiated in late-life in male mice. Using a multi-system approach (transcriptomic and metabolomics), this work illustrates the complexity of metformin’s physiological effects and underscores the need for a comprehensive assessment of the interaction that exists between metabolic dysfunction and aging phenotypic outcomes.

In addition to its involvement in lowering hepatic glucose production, metformin has recently been implicated as a direct regulator of metabolism, with actions including activation of a neuronal-mediated gut–brain–liver pathway,^37^ reduction in the rate of small intestinal glucose absorption to enhance postprandrial secretion of glucagon-like peptide-1,^38^ targeting of central carbon metabolism,^39^ anti-cancer protection,^40^ and the regulation of microRNA expression and cellular senescence.^41^ The present study demonstrates significant improvement in insulin sensitivity in non-fasted old mice subjected to intermittent metformin treatment for 17 weeks. This was evidenced by lower circulating insulin levels and HOMA-IR index, despite maintenance of blood glucose levels within the range seen with SD mice. This metformin protocol also reduced fasting plasma glucose and improved insulin sensitivity without translating into lifespan extension, even though limiting basal insulin levels in old mice has been recently reported to exert a positive effect on lifespan.^42^ We observed lower circulating leptin levels in the metformin-treated mice than in SD controls despite similar lean-to-fat ratio, supporting the idea that this drug is acting as a leptin sensitizer by increasing hepatic and hypothalamic leptin receptor expression.^43,44^

The rapid weight loss early in the metformin treatment could not be explained by the decrease in food intake or increase in foraging activity, but instead may have been the product of heightened rates of metabolic processes. Examination of the 24-month-old mice in the CLAMS monitoring system revealed the inability of metformin at promoting mitochondrial fatty acid oxidation during light cycle. Moreover, the removal of the drug during the intermittent metformin treatment was accompanied by a shift in carbohydrate utilization in both the light and dark cycles of EOW mice (RER values of 0.95 and 1.0, respectively), with a similar trend observed in the 2WM group. The greater reliance on sugar metabolism during the off-metformin period may be reflected by falling circulating lactate levels due to increased production of pyruvate and its ultimate conversion to ATP via aerobic metabolism through complex I of the respiratory chain.

The expression of hepatic genes involved in cholesterol biosynthesis, gluconeogenesis, and ribosome/translation were upregulated following chronic intermittent treatment of old mice with metformin for 17 weeks. Even though metformin has been shown to inhibit hepatic glucose production, a recent study using oral administration of glucose-1-^13^C in mice on high-fat diet reports on the increased conversion of labeled glucose to lactate-3-^13^C in the intestinal wall due to inhibition of complex I of the mitochondrial respiratory chain^7,45^ by metformin, and release of lactate-3-^13^C into the portal vein with subsequent generation of doubly labeled glucose-1,6-^13^C molecules in the liver via gluconeogenesis.^46^ The authors propose that this “futile” intestinal–zhepatic cycle is very energy consuming and could explain some of the reduced weight gain seen in HFD-fed mice on metformin. We reasoned that a similar mechanism could be implicated in the initial weight loss observed in old mice enrolled in the intermittent metformin program.

There are alterations in hepatic AMPK activity during normal aging in mice.^47^ It follows that major impact on hepatic lipid and glucose metabolism and energy production is expected when comparing young and old animals. Indeed, our preliminary data clearly showed that the daily cycling of energy expenditure over a 48-h period is markedly dampened with age, with RER values significantly higher during the light phase in 24-mo-old C57Bl/6 mice compared to 5-mo-old animals. These results are consistent with an age-associated substrate preference toward carbohydrate utilization for energy demand at the expense of fatty acid β-oxidation.

Because behavioral and motor coordination tests as well as the metabolic panel blood analyses were carried out at a single time point (e.g., 17 weeks of intermittent metformin treatment) instead of longitudinally, this study does not provide sufficient information to conclude that metformin promotes healthspan in aged mice. Despite the limitation of the study, several positive outcomes were observed in the liver of EOW mice, notably by histology and SEM, including improvement in the age-related changes in the porosity of the liver sieve.^28,48^ The integrity of the liver sieve is essential for the maintenance of insulin, glucose, and lipid metabolism and clearance of ingested and gut microbiota-derived metabolites.^49^ Aging predisposes the liver to chronic low-grade inflammation and oxidative damage that can contribute to the development of fibrosis and non-alcoholic fatty liver disease, which can evolve into non-alcoholic steatohepatitis in the absence of diet-induced obesity and type 2 diabetes.^50,51^ A recent study reported that the age-dependent increase in hepatic steatosis relates to cellular senescence.^52^ The ability of metformin to decrease cellular senescence and lower the abundance of inflammatory cytokines in several senescence models^41^ is in agreement with our findings that showed significant reduction in hepatic steatosis and liver injury in EOW mice. The failure to maintain homeostasis and adapt to environmental changes drives the pathogenesis of age-related disease of the liver. It is possible that some of the protection against hepatic inflammation in the aged EOW mice on metformin relates to the modulation of the gut microbiota through activation of the immune system.^53^ Clearly, more work is needed to validate this hypothesis.

Metformin-associated lactic acidosis is a known adverse side-effect of metformin therapy, which can be injurious in patients with renal dysfunction. Preclinical and clinical evidences show that the intestine appears to be an important source of metformin-induced lactate production^54,55^ and it typically emerges during meal absorption. This intestinal effect of metformin increases anaerobic metabolism of glucose to lactate and its utilization in the periphery. These observations are consistent with our basic serum metabolic panel showing the significant increase in circulating lactate in non-fasting EOW mice. At the time of sacrifice, the liver metabolome of EOW mice on metformin revealed a significant reduction in lactate, which may be the result of its increased conversion to pyruvate and cycling back to glucose via gluconeogenesis.^46^ Taken as a whole, these findings offer a promising use of metformin in combatting ailments of aging, although gastrointestinal intolerance, lactic acidosis, and kidney failure have contributed to the adverse side-effects associated with metformin use.^56^ Based on our results, it should be emphasized the importance of optimizing the schedule of metformin administration in order to gain the most health benefits.

Focusing on the molecular underpinnings of metformin action, transcriptomic and metabolomics analyses have been performed on liver tissue extracts from 2-year-old mice fed SD and mice on intermittent metformin protocol for 17 weeks. Microarray analysis illustrated the fact that EOW treatment impacted hepatic expression of several metabolically-relevant genes, including those encoding the transcriptional activator SREBF1—required for lipid homeostasis, the transcriptional coactivator PGC-1α—implicated in the regulation of genes involved in energy metabolism, and SOCS2—a negative regulator in the GH/IGF-1 signaling pathway. The increase in *Angptl4* expression in EOW livers is consistent with earlier reports showing the contribution of this liver-expressed serum hormone in the regulation of glucose homeostasis, lipid metabolism, and insulin sensitivity.^57^ Like metformin, long-term CR and exercise increase plasma ANGPTL4 levels in humans^58^ whereas decreased expression of *Angptl4* has been associated with type 2 diabetes.^57^ Lipin-1 plays a central role in mTORC1-dependent regulation of lipogenesis by SREBP1^59^ and we found that Lpin1 mRNA levels were significantly upregulated in EOW and 2WM livers together with an upregulation in pathways related to ribosome/translation. To what extent does the upregulation of genes encoding cholesterol and sterol biosynthetic enzymes and that of key proteins implicated in translation regulation antagonizes the pro-longevity effect of metformin remains to be determined. Noteworthingly, increases in lipoprotein cholesterol concentrations are associated with coronary atherosclerosis and shortened lifespan.^60^

Divergence between EOW and 2WM liver transcriptome was observed, notably with regard to GO Terms associated with mitochondrial inner membrane and mitochondrion, which could indicate the importance of optimizing the intermittent metformin treatment protocol for optimum benefits. We performed an exploratory data analysis aimed at visualizing the main characteristics of the current gene expression signatures with that of our recent CR study in male C57BL/6 J mice at 24 months of age.^32^ The analysis suggests great similarity in the gene expression profiles between CR and EOW, but not 2WM (Fig. 3j, k). Nevertheless, we observed differential regulation of lipid and cholesterol metabolism between metformin treatment and CR in these old mice.

Liver metabolomics analysis indicated the production of the oncometabolite 2-hydroxyglutarate from the tricarboxylic acid intermediate α-ketoglutarate. 2-Hydroxyglutarate is a known activator of mTOR through inhibition by the ternary complex between mTOR and the KDM4A-DEPTOR complex.^61^ KDM4A is an α-ketoglutarate-dependent enzyme of the Jumonji family of lysine demethylases that was recently found to act as a key regulator in the metabolic switch between mitochondrial oxidation and glycolysis,^62^ and to regulate senescence by targeting methylated p53 for degradation.^63^ Increase in nutrient availability promotes KDM4A-mediated ribosome biogenesis for the translation of RNA into protein,^64^ a very costly energy-consuming process that reduces life expectancy.^65^ It is possible that the inability of metformin to extend mouse lifespan when treatment was started late in life stemmed partly from the fact that 2-hydroxyglutarate levels were elevated with an upregulation of genes associated with ribosome biogenesis and assembly (Supplementary Table S4c). In contrast, inhibition of mTOR activity with concurrent activation of AMPK by rapamycin prolongs lifespan even when given in old mice.^66^ Therefore, metformin and rapalogs may exert cooperative effects at the level of the energy-sensing AMPK and diminished expenditure of cellular energy required for protein translation.

Tryptophan has been recently identified as a putative biomarker for type 2 diabetes risk^67^ and reduction in its intake appear to elicit beneficial health and lifespan effects.^68^ In earlier studies, tryptophan degradation into kynurenine intermediates and production of 3-indoxyl sulfate and related indoles such as indole-3-lactate have been found to elicit proinflammatory and adverse events on the cardiovascular and renal systems.^69,70^ Our metabolomics analysis revealed significant elevation in 3-indoxyl sulfate and/or indole-3-lactate in plasma of old EOW and 2WM mice, indicative of renal toxicity (Fig. 2i) with associated perturbation in glutathione biosynthesis and increased oxidative stress.^70^ Indeed, under metabolic stress conditions and with aging, the antioxidant defense system weakens and gives rise to a system-wide increase in pro-oxidative biomarker levels. Tissues such as the liver can dramatically increase the rate of hepatic glutathione synthesis by activating the transsulfuration pathway, which forms cystathionine from homocysteine, and release 2-hydroxybutanoic acid upon cleavage of cystathionine to cysteine. In time of need, glutamine is converted to glutamic acid, and along with glycine and cysteine, these amino acids are incorporated into glutathione. Herein, the marked increase in insulin sensitivity (lower HOMA-IR index) and significant elevation in plasma 2-hydroxybutanoic acid in old EOW mice was consistent with active transsulfuration pathway.

Hepatic conversion of glucose into fatty acids generates palmitoleic acid, a metabolite implicated in the regulation and pathophysiology of insulin action and glucose metabolism. A study on the relationship between palmitoleic acid and metabolic risk revealed that circulating palmitoleate is positively associated with greater adiposity, higher triglycerides, and greater insulin resistance in humans.^71^ Herein, EOW livers had significant reduction in palmitoleic acid, which is consistent with improved insulin sensitivity and lower hepatic triglyceride content.

To conclude, the metabolic effects of metformin EOW when initiated in late-life were associated with an overall improvement on health without an extension in lifespan as compared to control mice. A combination of confounding factors such as adiposity, energy overconsumption (ad libitum feeding) and related weight gain, mitochondrial dysfunction, and incidence of cancer could explain why metformin was unable to counterbalance these adverse life-shortening events despite exhibiting favorable markers of lower metabolic risk. Thus, additional longitudinal studies will need to carefully assess the effects of intermittent metformin treatment introduced at young to middle-age and how gender and rodent genetic background may modify or confound metformin’s effectiveness at improving healthspan, and possibly lifespan. Targeting lifespan extension is a very complex endeavor especially when a given intervention (e.g., metformin) is provided in late-life once evolutionarily conserved molecular pathways are already impaired, with genome stability loss, DNA repair defects, and chronic oxidative stress to name of few. The fact that metformin did not generate adverse outcomes in very old mice is a reassuring safety data possibly for humans that start metformin earlier in life and want to continue it into old age. Further focus on classical hallmarks of aging should provide new insights into whether/how improving healthspan can lead to lifespan extension.

## Materials and Methods

Full details are provided in the Supplementary Information section.

### Animals and diets

Animal procedures, housing and diets were in accordance with the guidelines issued by the Intramural Research Program of the National Institutes of Health protocol number 444TGB2016. Male C57BL/6 mice at 104 weeks of age were purchased from the National Institute on Aging Aged Rodent Colony from Charles River. The C57BL/6 mice maintained on a standard purified mouse diet (AIN-93G) for a month were randomly distributed to avoid differences in starting body weight average between the 3 experimental groups. Control group was kept on standard diet (SD; *n* = 68) until their death while the other groups of mice were fed SD plus 1% metformin following different on-and-off feeding protocols. Feedings protocol consist of every other week (EOW; *n* = 64) and two consecutive weeks each month (2WM; *n* = 67) with AIN-93G plus 1% metformin until their death. Sample size estimate to ensure adequate power to detect change in maximum lifespan was carried out based on our previous published work. Pure metformin was obtained from Farmhispania (Farmhispania S.A., Barcelona, Spain) and mixed to homogeneity during manufacturing of the diets (Dyets Inc., Bethlehem, PA). The chow was produced every 3 months during the length of the study, never was permitted to exceed 50 °C and was kept away from light whenever possible to ensure the stability of metformin (the light/dark cycle in the mouse facility was not altered). The mice were on a light:dark 12:12-h schedule and maintained between 20–22 °C with 30–70% relative humidity according to animal protocols and NIH guidelines. Food intake and body weight were measured on a biweekly basis for the duration of the study for control group and weekly for metformin fed mice. Body temperature was biweekly measured through an implantable temperature transponder system (IPTT-300, Bio Medic Data Systems). The transponders were implanted under the skin on the back of the animals between the shoulder blades using the provided instrument. The animals were allowed to heal and recover for a minimum of one week after the implantation before measurements were commenced. Survival curves were plotted using the Kaplan–Meier method, which includes all available animals at each time point. The criteria for euthanasia was based on an independent assessment by a veterinarian, according to AAALAC guidelines and only cases, where the condition of the animal was considered incompatible with continued survival, are represented in the curves. Animals removed at sacrifice for experimental procedures were considered as censored deaths.

The following tests in animals were performed by non-blinded investigators unless otherwise stated.

### Body composition

Measurements of lean, fat, and fluid mass in live mice were acquired by NMR using the Minispec LF90 (Bruker Optics, Billerica, MA, USA) after 16 weeks of treatment.

### Metabolic assessment

After 16 weeks of metformin treatment, mouse metabolic rate was assessed by indirect calorimetry in open-circuit Oxymax chambers with CLAMS (Columbus Instruments) as previously described.^2^ Movement (both horizontal and vertical) was also monitored with beams that software transforms into counts of beam breaks by the mouse.

### Physical performance tests

A detailed explanation of all physical performance tests performed is described in the Supplementary Information.

### Lactate and glucose measurements

Because diet was changed every week for EOW mice and every 2 consecutive weeks for 2WM mice, lactate and glucose levels were measured in 6-h fasted animals (*n* = 8–10) on the Friday of week 13, 14, 15, and 16 of treatment. Glucose and lactate concentrations in blood were measured by tail venipuncture with the Blood Glucose Monitoring System Breeze 2 (Bayer, Mishawaka, IN) and Lactate plus Meter (Nova Biomedical Corporation, Waltham, MA), respectively.

### Sample collection

After 17 weeks of treatment, a subgroup of non-fasted SD mice (*n* = 10) and mice on EOW and 2WM metformin (*n* = 6 each) were euthanized for experimental procedures. Glucose and lactate were measured from a drop of tail blood, with lactate measured from the first drop. Mice were anesthetized with ketamine–xylazine (50 mg kg^−1^ ketamine and 5 mg kg^−1^ xylazine; 10 µL 10 g^−1^ of body weight), and then blood and various tissues were collected as summarized in Supplementary Information.

### Blood and serum markers

An aliquot of whole blood was mixed with EDTA and used for the direct determination of HbA1c with an enzymatic kit (Crystal Chem, Downers Grove, IL). Coagulated whole blood was centrifuged at 12000×*g* for 10 min, and serum was aliquoted and kept at −80 °C. Serum leptin and adiponectin levels were measured using ELISA kits (EMD Millipore Corporation, Billerica, MA), and insulin levels were determined according to the manufacturer’s protocol (Crystal Chem).

### Histology and electron microscopy

Full methodological details for liver and kidney histology, and the determination of fenestrations in the liver sinusoidal endothelium by SEM can be found in Supplementary Information. Scoring was performed by investigators blinded to the treatment groups.

### Microarray analysis

Raw data were subjected to *Z*-normalization, as described elsewhere.^72,73^ PCA was performed on the normalized *Z*-scores of all of the detectable probes in the samples using DIANE 6.0 software, available from: (http://www.grc.nia.nih.gov/branches/rrb/dna/ diane_software.pdf). Significant genes were selected by the *z*-test < 0.05, false discovery rate < 0.30, as well as *z*-ratio > 1.5 in both directions and ANOVA *p* value < 0.05. Methodological details can be found in the Supplementary Information.

### Quantitative real-time PCR and Western blotting

Details can be found in the Supplementary Information.

### Metabolomics

Metabolomics analysis on mouse liver extracts and serum was performed by the UC Davis West Coast Metabolomics Center as previously described.^32^ Methodological details can be found in the Supplementary Information.

### Determination of hydrogen sulfide levels

The measure of hydrogen sulfide was carried out in liver extracts following the methodological details previously described.^26^ Quantification was performed by volume densitometry using ImageJ software.

### Determination of metformin levels

Method to measure metformin levels in mouse serum (*n* = 12) and liver homogenates (*n* = 14) was performed using a LC-MS/MS method developed at SRI International (Menlo Park, CA), as described in Supplementary Information. Lower limits of quantitation for the assay was 40 ng ml^−1^ in serum and 186 ng g^−1^ tissue in liver.

### Statistics

Equal variance and normal distribution tests were performed to select an appropriate statistical approach for each analysis. Unless otherwise stated, a non-parametric Kruskal–Wallis test followed by Dunn’s multiple comparisons test was used. For longevity studies, Gehan–Breslow-Wilcoxon statistical test was used. Analyses were performed using GraphPad Prism v. 6 (GraphPad Software, Inc., La Jolla, CA). In all experiments, results are represented as the mean ± SEM, with *p* values < 0.05 considered significant.

### Data availability

Microarray data have been deposited in the Gene Expression Omnibus database under accession code GSE97074. All relevant data that support the findings of this study are included in this published article (and its Supplementary information files), or are available from the corresponding authors on reasonable request.

## Electronic supplementary material

Supplemental Material

## Electronic supplementary material

**Supplementary information** accompanies the paper on the *npj Aging and Mechanisms of Disease* website (10.1038/s41514-017-0018-7).

## References

[1] US Food and Drug Administration. *Metformin Information* https://www.fda.gov/Drugs/DrugSafety/PostmarketDrugSafetyInformationforPatientsandProviders/ucm493293.htm (2017).

[2] Martin-Montalvo A (2013). Metformin improves healthspan and lifespan in mice. Nat. Commun..

[3] Anisimov VN (2011). If started early in life, metformin treatment increases life span and postpones tumors in female SHR mice. Aging (Albany NY).

[4] Strong R (2016). Longer lifespan in male mice treated with a weakly estrogenic agonist, an antioxidant, an α-glucosidase inhibitor or a Nrf2-inducer. Aging Cell.

[5] De Haes W (2014). Metformin promotes lifespan through mitohormesis via the peroxiredoxin PRDX-2. Proc. Natl. Acad. Sci. USA.

[6] Cabreiro F (2013). Metformin retards aging in C. elegans by altering microbial folate and methionine metabolism. Cell.

[7] Owen MR, Doran E, Halestrap AP (2000). Evidence that metformin exerts its anti-diabetic effects through inhibition of complex 1 of the mitochondrial respiratory chain. Biochem. J..

[8] El-Mir MY (2000). Dimethylbiguanide inhibits cell respiration via an indirect effect targeted on the respiratory chain complex I. J. Biol. Chem..

[9] Madiraju AK (2014). Metformin suppresses gluconeogenesis by inhibiting mitochondrial glycerophosphate dehydrogenase. Nature.

[10] Kalender A (2010). Metformin, independent of AMPK, inhibits mTORC1 in a rag GTPase-dependent manner. Cell. Metab..

[11] Takiyama Y (2011). Tubular injury in a rat model of type 2 diabetes is prevented by metformin: a possible role of HIF-1α expression and oxygen metabolism. Diabetes.

[12] Chen SC (2017). Metformin suppresses adipogenesis through both AMP-activated protein kinase (AMPK)-dependent and AMPK-independent mechanisms. Mol. Cell Endocrinol..

[13] Foretz M (2010). Metformin inhibits hepatic gluconeogenesis in mice independently of the LKB1/AMPK pathway via a decrease in hepatic energy state. J. Clin. Invest..

[14] Wu L (2016). An ancient, unified mechanism for metformin growth inhibition in C. elegans and cancer. Cell.

[15] Vazquez-Martin A, Oliveras-Ferraros C, Menendez JA (2009). The antidiabetic drug metformin suppresses HER2 (erbB-2) oncoprotein overexpression via inhibition of the mTOR effector p70S6K1 in human breast carcinoma cells. Cell Cycle.

[16] Vazquez-Martin A (2012). Metformin limits the tumourigenicity of iPS cells without affecting their pluripotency. Sci. Rep..

[17] López-Otín C, Blasco MA, Partridge L, Serrano M, Kroemer G (2013). The hallmarks of aging. Cell.

[18] Menendez JA (2011). Metformin and the ATM DNA damage response (DDR): accelerating the onset of stress-induced senescence to boost protection against cancer. Aging (Albany NY).

[19] Novelle MG, Ali A, Diéguez C, Bernier M, de Cabo R (2016). Metformin: a hopeful promise in aging research. Cold Spring Harb. Perspect. Med..

[20] Smith DL (2010). Metformin supplementation and life span in Fischer-344 rats. J. Gerontol. A. Biol. Sci. Med. Sci..

[21] Onken B, Driscoll M (2010). Metformin induces a dietary restriction-like state and the oxidative stress response to extend C. elegans Healthspan via AMPK, LKB1, and SKN-1. PLoS One.

[22] Barzilai N, Crandall JP, Kritchevsky SB, Espeland MA (2016). Metformin as a tool to target aging. Cell Metab..

[23] Reagan-Shaw S, Nihal M, Ahmad N (2008). Dose translation from animal to human studies revisited. FASEB J..

[24] DeFronzo R, Fleming GA, Chen K, Bicsak TA (2016). Metformin-associated lactic acidosis: current perspectives on causes and risk. Metabolism.

[25] Perry RJ, Samuel VT, Petersen KF, Shulman GI (2014). The role of hepatic lipids in hepatic insulin resistance and type 2 diabetes. Nature.

[26] Hine C (2015). Endogenous hydrogen sulfide production is essential for dietary restriction benefits. Cell.

[27] Kim IH, Kisseleva T, Brenner DA (2015). Aging and liver disease. Curr. Opin. Gastroenterol..

[28] Mohamad M (2016). Ultrastructure of the liver microcirculation influences hepatic and systemic insulin activity and provides a mechanism for age-related insulin resistance. Aging Cell.

[29] Finck BN (2006). Lipin 1 is an inducible amplifier of the hepatic PGC-1α/PPARα regulatory pathway. Cell Metab..

[30] Guillén N (2009). Microarray analysis of hepatic gene expression identifies new genes involved in steatotic liver. Physiol. Genomics.

[31] Zhou Y, Jiang L, Rui L (2009). Identification of MUP1 as a regulator for glucose and lipid metabolism in mice. J. Biol. Chem..

[32] Mitchell SJ (2016). Effects of sex, strain, and energy intake on hallmarks of aging in mice. Cell Metab..

[33] Knopf JL, Gallagher JF, Held WA (1983). Differential, multihormonal regulation of the mouse major urinary protein gene family in the liver. Mol. Cell Biol..

[34] Kang HS (2016). Metformin stimulates IGFBP-2 gene expression through PPARalpha in diabetic states. Sci. Rep..

[35] Kang HS (2015). Regulation of IGFBP-2 expression during fasting. Biochem. J..

[36] Leiser SF (2015). Cell nonautonomous activation of flavin-containing monooxygenase promotes longevity and health span. Science.

[37] Duca FA (2015). Metformin activates a duodenal Ampk-dependent pathway to lower hepatic glucose production in rats. Nat. Med..

[38] Wu T (2017). Metformin reduces the rate of small intestinal glucose absorption in type 2 diabetes. Diabetes Obes. Metab..

[39] Liu X, Romero IL, Litchfield LM, Lengyel E, Locasale JW (2016). Metformin targets central carbon metabolism and reveals mitochondrial requirements in human cancers. Cell. Metab..

[40] Benjamin D (2016). Syrosingopine sensitizes cancer cells to killing by metformin. Sci. Adv..

[41] Noren Hooten N (2016). Metformin-mediated increase in DICER1 regulates microRNA expression and cellular senescence. Aging Cell.

[42] Templeman NM (2017). Reduced circulating insulin enhances insulin sensitivity in old mice and extends lifespan. Cell Rep..

[43] Tang X (2016). Metformin increases hepatic leptin receptor and decreases steatosis in mice. J. Endocrinol..

[44] Aubert G, Mansuy V, Voirol MJ, Pellerin L, Pralong FP (2011). The anorexigenic effects of metformin involve increases in hypothalamic leptin receptor expression. Metabolism.

[45] Bridges HR, Jones AJ, Pollack MN, Hirst J (2014). Effects of metformin and other biguanides on oxidative phosphorylation in mitochondria. Biochem. J..

[46] Schommers P (2017). Metformin causes a futile intestinal-hepatic cycle which increases energy expenditure and slows down development of a type 2 diabetes-like state. Mol. Metab..

[47] Mulligan JD, Gonzalez AA, Kumar R, Davis AJ, Saupe KW (2005). Aging elevates basal adenosine monophosphate-activated protein kinase (AMPK) activity and eliminates hypoxic activation of AMPK in mouse liver. J. Gerontol. A Biol. Sci. Med. Sci..

[48] Jamieson HA (2007). Caloric restriction reduces age-related pseudocapillarization of the hepatic sinusoid. Exp. Gerontol..

[49] Cogger VC (2016). Dietary macronutrients and the aging liver sinusoidal endothelial cell. Am. J. Physiol. Heart Circ. Physiol..

[50] Fontana L (2013). Aging promotes the development of diet-induced murine steatohepatitis but not steatosis. Hepatology.

[51] S. Jiang (2017). AMPK orchestrates an elaborate cascade protecting tissue from fibrosis and aging. Ageing Res. Rev..

[52] Ogrodnik M (2017). Cellular senescence drives age-dependent hepatic steatosis. Nat. Commun..

[53] Neyrinck, A. M. et al. Spirulina protects against hepatic inflammation in aging: an effect related to the modulation of the gut microbiota? *Nutrients*10.3390/nu9060633 (2017). 10.3390/nu9060633 PMC5490612 28632181

[54] Pénicaud L, Hitier Y, Ferré P, Girard J (1989). Hypoglycaemic effect of metformin in genetically obese (fa/fa) rats results from an increased utilization of blood glucose by intestine. Biochem. J..

[55] Bailey CJ, Wilcock C, Scarpello JHB (2008). Metformin and the intestine. Diabetologia.

[56] McCreight LJ, Bailey CJ, Pearson ER (2016). Metformin and the gastrointestinal tract. Diabetologia.

[57] Xu A (2005). Angiopoietin-like protein 4 decreases blood glucose and improves glucose tolerance but induces hyperlipidemia and hepatic steatosis in mice. Proc. Natl. Acad. Sci. USA.

[58] Kersten S (2009). Caloric restriction and exercise increase plasma ANGPTL4 levels in humans via elevated free fatty acids. Arterioscler. Thromb. Vasc. Biol..

[59] Peterson TR (2011). mTOR complex 1 regulates lipin 1 localization to control the SREBP pathway. Cell.

[60] Verbeek R, Hovingh GK, Boekholdt SM (2015). Non-high-density lipoprotein cholesterol: current status as cardiovascular marker. Curr. Opin. Lipidol..

[61] Carbonneau M (2016). The oncometabolite 2-hydroxyglutarate activates the mTOR signalling pathway. Nat. Commun..

[62] Wang LY (2016). KDM4A Coactivates E2F1 to Regulate the PDK-Dependent Metabolic Switch between Mitochondrial Oxidation and Glycolysis. Cell Rep..

[63] Johmura Y (2016). SCF(Fbxo22)-KDM4A targets methylated p53 for degradation and regulates senescence. Nat. Commun..

[64] Salifou K (2016). The histone demethylase JMJD2A/KDM4A links ribosomal RNA transcription to nutrients and growth factors availability. Nat. Commun..

[65] Hofmann JW (2015). Reduced expression of MYC increases longevity and enhances healthspan. Cell.

[66] Harrison DE (2009). Rapamycin fed late in life extends lifespan in genetically heterogeneous mice. Nature.

[67] Chen T (2016). Tryptophan predicts the risk for future type 2 diabetes. PLoS One.

[68] van der Goot AT, Nollen EA (2013). Tryptophan metabolism: entering the field of aging and age-related pathologies. Trends Mol. Med..

[69] Barisione C (2015). Indoxyl sulfate: a candidate target for the prevention and treatment of cardiovascular disease in chronic kidney disease. Curr. Drug Targets.

[70] Yu M, Kim YJ, Kang DH (2011). Indoxyl sulfate-induced endothelial dysfunction in patients with chronic kidney disease via an induction of oxidative stress. Clin. J. Am. Soc. Nephrol..

[71] Mozaffarian D (2010). Circulating palmitoleic acid and risk of metabolic abnormalities and new-onset diabetes. Am. J. Clin. Nutr..

[72] Cheadle C, Vawter MP, Freed WJ, Becker KG (2003). Analysis of microarray data using Z score transformation. J. Mol. Diagn..

[73] Lee JS (2012). Meta-analysis of gene expression in the mouse liver reveals biomarkers associated with inflammation increased early during aging. Mech. Ageing Dev..

[74] Hilmer SN (2004). The effect of aging on the immunohistochemistry of apolipoprotein E in the liver. Exp. Gerontol..

[75] Kim SY, Volsky DJ (2005). PAGE: parametric analysis of gene set enrichment. Bmc. Bioinformatics.

