# Supplementary material for: Health benefits of late-onset metformin treatment every other week in mice
Source: NPJ Aging Mech Dis. 2017 Nov 20;3:16. doi: 10.1038/s41514-017-0018-7 (PMC5696465; doi:10.1038/s41514-017-0018-7)
Supplement: Supplementary file 1 — Supplemental Material [file 41514_2017_18_MOESM1_ESM.pdf]

Supplementary Information

Manuscript # NPJAMD-00130R
Alfaras I. *et al.* Health benefits of late-onset metformin treatment every other week in mice

Index ……………………………………………..…………………………………………..……. 1
Supplementary Figures and associated Legends ……………………………………………………. 2-4

Figure S1. **Physiological data**. Effect of metformin on physical performance, metabolically relevant biomarkers, and hepatic hydrogen sulfide production in mice.

Figure S2. **Liver microarray data and AMPK activation levels at steady state.**

Supplementary Tables ………………………………………………………………………………… 5-15

Table S1. Survival statistics for study mice stratified by metformin treatment groups.

Table S2. Gross histopathology analysis of tissues collected at death or euthanasia.

Table S3. List of the top 50 significantly enriched genes shared in the livers of EOW-SD and 2WM-SD pairwise comparisons.

Table S4a. List of the 23 significantly enriched GO Terms in EOW livers.

Table S4b. List of the top significantly enriched GO Terms in 2WM livers

Table S4c. List of the 45 significantly enriched GO Terms shared in the livers of EOW-SD and 2WM-SD pairwise comparisons.

Table S5a. List of significant genes shared in the livers of 2WM-SD and CR40-AL pairwise comparisons.

Table S5b. List of significant genes shared in the livers of EOW-SD and CR40-AL pairwise comparisons.

Table S5c. List of significant genes shared in the livers of EOW-SD, 2WM-SD, and CR40-AL pairwise comparisons.

Table S6a. List of significant liver metabolites in the EOW-SD and 2WM-SD pairwise comparisons.

Table S6b. List of significant serum metabolites in the EOW-SD and 2WM-SD pairwise comparisons.

Supplementary Methods ………………………………………………………………………….. 16-20

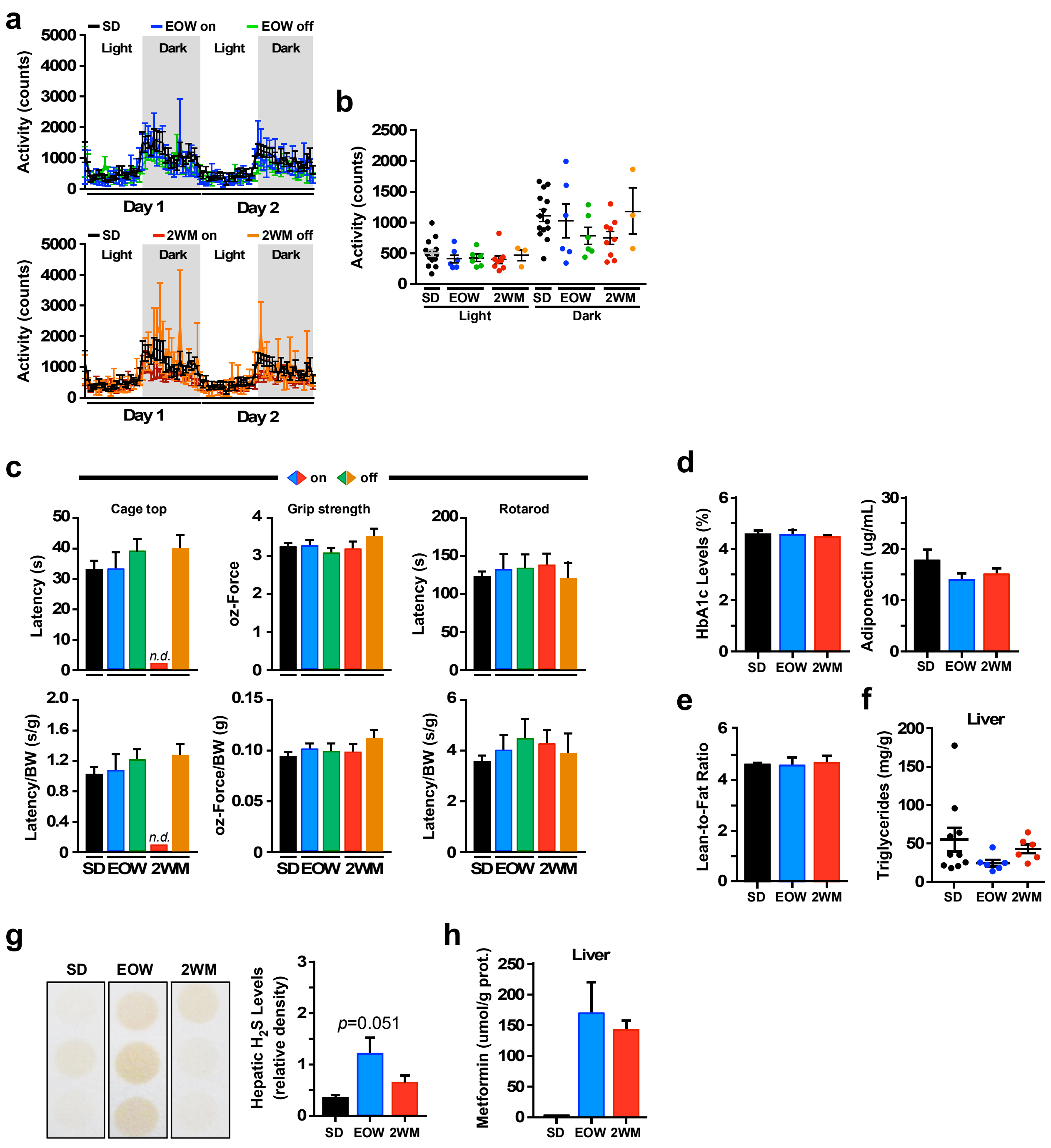

Figure S1. Effect of metformin on physical performance, metabolically relevant biomarkers, and hepatic hydrogen sulfide production in mice. (**a**) Activity measures of mice put in metabolic chambers for 48 h (SD, *n*=11; EOW on metformin, *n*=6; EOW off metformin, *n*=6; 2WM on metformin, *n*=9; 2WM off metformin, *n*=3). (**b**) Activity counts shown in **b** were segregated based on the light and dark periods of the L12:D12 cycle. (**c**) Latency to fall from elevated cage top or rotarod and grip strength were measured after 16 weeks of treatment (for cage top: SD, *n*=47; EOW on metformin, *n*=12; EOW off metformin, *n*=7; 2WM on metformin, *n*=0; 2WM off metformin, *n*=22); for both grip strength and rotarod: SD, *n*=43; EOW

on metformin, *n*=7; EOW off metformin, *n*=11; 2WM on metformin, *n*=14; 2WM off metformin, *n*=9). **Upper panels**, before and **lower panels**, after correction for body weight. (**d**) Levels of blood HbA1c and serum adiponectin in fed mice (SD, *n*=10; EOW, *n*=6; 2WM, *n*=6). (**e**) Lean-to-fat ratio after 16 weeks on diet (SD, *n*=61; EOW, *n*=18; 2WM, *n*=21). (**f**) Levels of triglycerides in liver extracts (SD, *n*=10; EOW, *n*=6; 2WM, *n*=6). (**g**) Production of H_2S in total liver lysates of fasted mice (SD, *n*=10; EOW, *n*=6; 2WM, *n*=6). Data are represented as the mean ± s.e.m. (**h**) Hepatic levels of metformin after 17 weeks of treatment (SD, *n*=3; EOW, *n*=6; 2WM, *n*=6). Data are represented as the mean ± s.e.m. SD, standard diet.

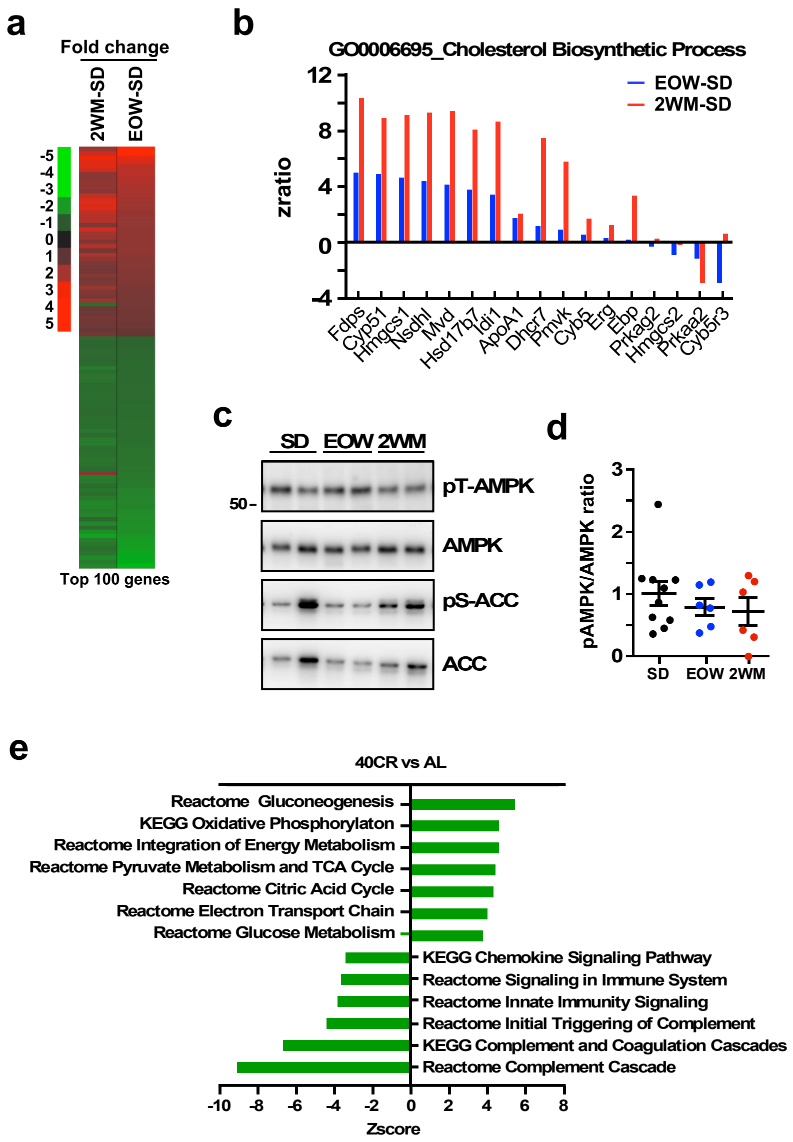

Figure S2. Liver microarray data and AMPK activation levels at steady state. (a) Heat map comparing the top 100 liver transcripts significantly upregulated (red) and downregulated (green) in mice on EOW and 2WM diets. (**b**) Expression profile of genes enriched in the GO Term 'Cholesterol Biosynthetic Process' in the indicated pairwise comparisons. **(c)** Western blots of liver extracts of mice on SD, EOW or 2WM metformin treatment for 17 weeks. The signal associated with phosphorylated AMPK (pThr-172) and ACC (pSer-79) and their total forms is depicted in these representative autoradiograms. **(d)** Densitometric analysis of western blots in (**c**) was used to calculate the phosphorylated/total AMPK and ACC ratios. Means ± s.e.m. are shown. SD, n=10; EOW, n=6; 2WM, n=6. (**e**) Liver microarray data from our recent study on the chronic effect of 40% CR versus *ad libitum* feeding (CR40-AL) in male C57BL/6J mice[32]. Data are represented as the mean ± s.e.m; n=6 biological replicates per group, 23-24 months of age, 17-18 months on diet. Partial list of top hepatic gene sets significantly changed in the CR40-AL pairwise comparison is depicted.

Table S1, related to Figure 1b. Survival statistics for study mice stratified by metformin treatment group

	**Survival (weeks)**					
**Group (# mice)**	**Q1**	**Mean**	**Median**	**Q3**	**90% (max)**	**Max**
SD (68)	122.4	132.4	131.4	141.9	149.3	167.9
EOW (64)	118.7 (-3)	130.8 (-1)	127.9 (-3)	144.1 (+2)	151.0 (+1)	157.0 (-6)
2WM (67)	114.2 (-7)	125.8 (-5)	123.1 (-6)	134.9 (-5)	145.6 (-2)	163.6 (-3)

The survival for EOW and 2WM groups is represented in weeks with the mean percentage change in survival relative to the SD group indicated in the parentheses. SD, standard diet; EOW, metformin treatment every-other-week; 2WM, metformin treatment for two consecutive weeks each month.

Table S2, related to Figure 1b. Gross histopathology analysis of tissues collected at death or euthanasia

		**Experimental Group (number of mice)**		
		SD (48)	EOW (50)	2WM (52)
Mean age of death (weeks)		132 ± 2	131 ± 2	126 ± 2
Wet body	N	1	2	2
Kyphosis	N	43	43	49
Loss of fur color	N	39	40	44
Thymus	Enlarged	5	3	3
Heart	Enlarged	16	15	19
	Ischemic change	25	18	14
Lungs	Cancer/mass	6	5	2
	Dark red/bloody	15	16	14
	Pale/pink	0	0	1
Spleen	Enlarged	19	17	11
	Pale/dark/motled	18	19	12
	Mass/tumor/cancer	4	2	2
Liver	Enlarged	26	23	26
	Cirrhosis/cancer	13	13	15
	Pale/fatty	12	9	9
Kidneys	Enlarged	12	9	10
	Discolored	15	19	22
	Misshapen	3	4	5
Abdominal cavity	Mass/tumor	7	5	5
Testes	Mass/tumor	1	0	0
Neck	Mass/tumor	0	0	1

SD, standard diet; EOW, metformin treatment every-other-week; 2WM, metformin treatment for two consecutive weeks each month.

Table S3, related to Figure 3b. List of the top 50 significantly enriched genes shared in the livers of EOW-SD and 2WM-SD pairwise comparisons

Symbol	EOW-SD zratio	2WM-SD zratio	Symbol	EOW-SD zratio	2WM-SD zratio
Lpin1	11.31	6.82	EG624219	-5.90	-7.51
Sqle	8.05	12.29	Abca8a	-5.78	-3.98
Pck1	7.70	9.07	1300013D18Rik	-5.77	-4.65
Sc4mol	6.37	10.50	Rtp3	-5.62	-3.68
1810055G02Rik	5.46	6.18	1600023A02Rik	-5.59	-4.77
Lpin2	5.19	4.76	Mreg	-5.49	-5.17
Trib3	5.11	5.53	Es22	-5.43	-3.26
Gpnmb	4.97	4.87	Gck	-5.00	-5.34
Fdps	4.92	10.26	Cyp2d13	-4.72	-3.29
Cyp51	4.85	8.89	9030624L02Rik	-4.58	-2.83
Got1	4.62	4.80	Pycrl	-4.50	-3.28
Hmgcs1	4.61	9.09	Inhba	-4.33	-5.52
Zfp259	4.23	2.27	Saa4	-4.25	-5.26
Mvd	4.12	9.31	Acbd5	-4.17	-4.88
Rapgef4	3.86	3.75	Il1rap	-3.89	-2.18
Hsd17b7	3.82	8.02	Ppp1r3b	-3.77	-5.11
BC036718	3.71	2.71	Tcea3	-3.65	-1.98
Lss	3.68	7.94	Tmem32	-3.58	-1.88
Sult1a1	3.66	2.21	Tgoln1	-3.56	-3.80
Irs2	3.64	3.67	C730031G17	-3.48	-2.10
Rpl3	3.57	2.44	Dhrs8	-3.16	-3.08
Nsdhl	3.54	7.67	Acy3	-3.09	-4.08
Idi1	3.40	8.63	Slc17a3	-3.08	-2.68
3010033P07Rik	3.37	2.48	1110057K04Rik	-3.08	-2.63
Nfe2	3.29	5.54	Klkb1	-3.06	-2.26

The zratios of significantly up- (red font) and down- (blue font) regulated genes in each pairwise comparison are shown. Significance is defined as zratio > 1.5 in either direction, false discovery rate (fdr) < 0.3 and p < 0.05. N = 4 mice per experimental group. EOW, metformin treatment every-other-week; 2WM, metformin treatment for two consecutive weeks each month; SD, standard diet.

Table S4a, related to Figure 3c. List of the 23 significantly enriched GO Terms in EOW livers

GO Term	(n)	EOW-SD Z-score
GO0007155 Cell Adhesion	469	4.209
GO0005507 Copper Ion Binding	63	4.113
GO0006707 Cholesterol Catabolic Process	7	3.262
GO0016064 Immunoglobulin Mediated Immune Response	16	2.950
GO0008199 Ferric Iron Binding	11	2.449
GO0000149 Snare Binding	6	2.039
GO0015057 Thrombin Receptor Activity	6	2.007
GO0006836 Neurotransmitter Transport	35	1.602
GO0016581 Nurd Complex	7	-1.973
GO0046875 Ephrin Receptor Binding	7	-2.634
GO0006512 Ubiquitin Cycle	310	-2.755
GO0006888 ER to Golgi Vesicle Mediated Transport	61	-2.787
GO0030122 AP2 Adaptor Complex	6	-2.882
GO0008565 Protein Transporter Activity	139	-2.913
GO0005778 Peroxisomal Membrane	24	-3.217
GO0016874 Ligase Activity	221	-3.248
GO0017111 Nucleoside Triphosphatase Activity	109	-3.277
GO0006886 Intracellular Protein Transport	199	-3.312
GO0042802 Identical Protein Binding	125	-3.736
GO0007031 Peroxisome Organization and Biogenesis	23	-3.817
GO0008152 Protein Transport	396	-3.874
GO0008152 Metabolic Process	487	-4.468
GO0006633 Fatty Acid Biosynthetic Process	40	-5.604

Table S4b, related to Figure 3c. List of the top significantly enriched GO Terms in 2WM livers

GO Term	(n)	2WM-SD Z-score
GO0006694 Steroid Biosynthetic Process	47	16.945
GO0008610 Lipid Biosynthetic Process	79	13.913
GO0016491 Oxidoreductase Activity	542	4.981
GO0002474 Antigen Processing and Presentation of Peptide Antigen ..	10	4.832
GO0005839 Proteasome Core Complex (Sensu Eukaryota)	19	4.486
GO0004298 Threonine Endopeptidase Activity	18	4.392
GO0008137 NADH Dehydrogenase (Ubiquinone) Activity	34	4.013
GO0004866 Endopeptidase Inhibitor Activity	93	3.843
GO0003954 NADH Dehydrogenase Activity	15	3.596
GO0005550 Pheromone Activity	95	3.343
GO0016568 Chromatin Modification	116	-3.145
GO0005096 GTPase Activator Activity	130	-3.146
GO0005515 Protein Binding	3771	-3.618
GO0008270 Zinc Ion Binding	1471	-3.726
GO0004674 Protein Serine or Threonine Kinase Activity	357	-3.752
GO0006350 Transcription	1204	-4.317
GO0006355 Regulation of Transcription DNA Dependent	1486	-4.459

Table S4c, related to Figure 3d. List of the 45 significantly enriched GO Terms shared in the livers of EOW-SD and 2WM-SD pairwise comparisons

GO Term	(n)	EOW-SD Z-score	2WM-SD Z-score
GO0005830 Cytosolic Ribosome (Sensu Eukaryota)	29	9.797	4.334
GO0042254 Ribosome Biogenesis and Assembly	78	7.282	3.533
GO0005578 Proteinaceous Extracellular Matrix	278	7.029	6.806
GO0006695 Cholesterol Biosynthetic Process	19	7.027	19.939
GO0005840 Ribosome	125	6.875	4.871
GO0016126 Sterol Biosynthetic Process	17	6.619	20.953
GO0003735 Structural Constituent of Ribosome	136	6.596	4.191
GO0005576 Extracellular Region	539	6.165	4.317
GO0005615 Extracellular Space	1864	5.999	5.051
GO0006412 Translation	217	5.274	4.071
GO0007165 Signal Transduction	2212	4.873	3.529
GO0030529 Ribonucleoprotein complex	218	4.789	3.591
GO0019843 RRNA Binding	14	4.562	2.414
GO0005843 Cytosolic Small Ribosome Subunit (Sensu Eukaryota)	11	4.430	3.458
GO0042157 Lipoprotein Metabolic Process	13	4.396	4.718
GO0009897 External Side of Plasma Membrane	111	4.206	4.376
GO0004871 Signal Transducer Activity	1711	4.167	4.258
GO0004872 Receptor Activity	2493	3.754	3.760
GO0006817 Phosphate Transport	63	3.681	3.973
GO0007186 G Protein Coupled Receptor Protein Signaling	1600	3.674	4.396
GO0004930 G Protein Coupled Receptor Activity	1486	3.653	4.681
GO0005201 Extracellular Matrix Structural Constituent	72	3.620	3.400
GO0030020 Extracellular Matrix Structural Constituent Confer...	27	3.306	2.775
GO0005604 Basement Membrane	47	3.277	2.754
GO0007608 Sensory Perception of Smell	1084	3.179	4.586
GO0004984 Olfactory Receptor Activity	1070	3.152	4.667
GO0017127 Cholesterol Transporter Activity	8	3.137	3.265
GO0048503 GPI Anchor Binding	126	2.960	3.972
GO0006955 Immune Response	342	2.903	6.644
GO0005581 Collagen	30	2.900	2.106
GO0006541 Glutamine Metabolic Process	10	2.792	2.967
GO0006952 Defense Response	167	2.150	4.121
GO0001584 Rhodopsin Like Receptor Activity	453	1.741	2.816
GO0009072 Aromatic Amino Acid Family Metabolic Process	13	1.595	2.340
GO0006506 GPI Anchor Biosynthetic Process	18	-1.642	-2.082
GO0016573 Histone Acetylation	9	-1.738	-3.413
GO0000776 Kinetochore	24	-2.020	-2.200
GO0030041 Actin Filament Polymerization	11	-2.106	-3.470
** GO0005786 Signal Recognition Particle Endoplasmic	8	-2.374	1.749
GO0030032 Lamellipodium Biogenesis	16	-2.473	-2.326
** GO0005743 Mitochondrial Inner Membrane	214	-3.332	5.023
GO0005794 Golgi Apparatus	508	-3.538	-2.273
GO0007001 Chromosome Organization and Biogenesis	81	-3.864	-2.979
GO0006334 Nucleosome Assembly	83	-4.020	-2.854
** GO0005739 Mitochondrion	775	-4.703	3.244

The Z-scores of significantly up- (red font) and down- (blue font) regulated GO Terms in a given pairwise comparison are shown. Significance is defined as Z-score > 1.5 in either direction, false discovery rate (fdr) < 0.3 and $p < 0.05$. N = 4 mice per experimental group. EOW, metformin treatment every-other-week; 2WM, metformin treatment for two consecutive weeks each month; SD, standard diet; n, number of genes in a given GO Term.

** GO Terms that follow opposite direction between EOW-SD and 2WM-SD pairwise comparisons.

Table S5a, related to Figure 3k. List of significant genes shared in the livers of 2WM and CR40 mice

	2WM-SD	CR40-AL		2WM-SD	CR40-AL
**Symbol**	**zratio**	**zratio**	**Symbol**	**zratio**	**zratio**
Atp5a1	3.07	3.52	Bach1	-4.17	-2.11
Gch1	2.38	2.71	Zbtb7	-3.36	-3.65
Hspa9	1.61	2.65	F7	-2.81	-1.50
			Atp8b1	-2.51	-2.33
2510004L01Rik	11.38	-4.10	Lrrc3	-2.21	-2.71
G1p2	10.77	-3.05	2610003J06Rik	-2.20	-2.39
Oasl2	10.57	-1.70			
Ly6a	10.34	-3.26	Per2	-4.87	6.18
Gvin1	8.28	-2.65	Abcg5	-4.46	4.47
Rdh11	7.95	-2.71	BC011209	-3.41	3.46
D12Ertd647e	7.44	-4.35	Epha1	-3.35	2.71
Dhcr7	7.41	-2.79	Dnajb1	-3.32	2.40
2310061N23Rik	7.25	-5.54	Tmem20	-2.98	3.04
Stat1	6.72	-1.76	Spg4	-2.98	2.86
Ifi47	6.41	-3.60	5730578N08Rik	-2.76	4.31
Ly6e	6.24	-5.17	1700048E23Rik	-2.64	2.61
Aqp8	6.21	-11.33	BC016495	-2.58	2.98
Acly	5.34	-4.34	9530058B02Rik	-2.54	2.54
Gale	5.22	-3.63	Abcf2	-2.46	2.13
Ube1l	4.88	-2.39	Nasp	-2.42	1.55
S100a10	4.48	-5.14	2310037I24Rik	-2.40	1.53
AA175286	4.13	-3.14	4932441K18Rik	-2.37	2.84
Lgals3bp	4.12	-3.15	Herpud1	-2.34	2.52
Pgd	3.65	-2.96	2010319C14Rik	-2.16	3.17
Ifitm3	3.42	-3.20	Gkap1	-2.15	1.72
Ube2l6	3.34	-2.35	Adssl1	-2.01	2.39
Elovl5	3.20	-3.94	D7Rp2e	-1.96	3.60
Tars	3.05	-1.67	3300001P08Rik	-1.93	1.57
Tuba6	2.89	-4.08	Mir16	-1.83	4.57
1300014I06Rik	2.63	-1.99	Sertad2	-1.75	1.98
2310008M10Rik	2.63	-4.38	Chchd7	-1.64	3.39
Rnf125	2.30	-5.54	Man2a1	-1.55	2.24
Ifi35	2.26	-1.57			
Sec22b	2.21	-2.93			
Itpk1	2.19	-1.85			
1810044O22Rik	2.14	-2.62			
Mocos	2.05	-1.61			
Sec61b	1.90	-1.72			
Pcbd	1.81	-2.65			
Tuba1a	1.58	-2.35			

Table S5b, related to Figure 3k. List of significant genes shared in the livers of EOW and CR40 mice

	EOW-SD	CR40-AL		EOW-SD	CR40-AL
Symbol	zratio	zratio	Symbol	zratio	zratio
Fmo3	20.36	24.89	Mup5	-14.59	-12.07
1700018018Rik	8.93	5.56	OTTMUSG00..07485	-14.21	-13.72
Angptl4	7.57	4.78	OTTMUSG0..000231	-13.35	-13.20
Socs2	6.80	5.88	Car3	-12.61	-10.95
Por	6.51	9.22	2810007J24Rik	-11.49	-7.89
Alas1	6.47	5.15	LOC620807	-8.68	-20.64
Cyp2a5	6.14	10.96	Dio1	-8.51	-7.77
Nt5e	5.30	2.25	Srd5a1	-6.19	-4.12
Rbm3	4.91	5.37	Keg1	-6.13	-6.18
Arrdc4	4.82	6.20	Mup1	-6.05	-17.59
Slco1a4	4.66	9.32	Mup2	-5.59	-24.14
Asl	4.63	4.46	Slc30a10	-5.55	-4.26
Rdh9	4.55	7.37	C8a	-5.36	-5.58
Tef	4.46	7.12	Inhbc	-5.28	-2.48
Siat9	4.35	4.19	Ociad2	-5.13	-3.03
Pnpla2	4.32	5.90	Ugt3a1	-5.10	-9.37
Rpl12	4.13	2.17	Cml1	-4.73	-4.75
Plk3	4.05	4.49	BC004728	-4.64	-3.25
Ldb1	3.98	4.99	1700019G17Rik	-4.52	-5.49
Aprt	3.93	3.07	Srebf1	-4.37	-4.27
Ppargc1a	3.76	2.42	Cml2	-4.36	-2.92
Cd63	3.40	1.76	Mug2	-4.19	-4.67
Scara3	3.35	2.37	Serpina12	-4.12	-13.92
BC011468	3.31	2.75	Paqr9	-4.10	-5.15
Amid	3.12	3.08	9130214H05Rik	-3.98	-2.39
Ccbl2	3.00	2.86	Hdhd3	-3.71	-2.21
Cdkn1c	2.98	5.01	Mbl1	-3.69	-3.87
Mdh2	2.83	5.69	Efcbp1	-3.58	-2.13
Rpl5	2.74	2.11	Cldn1	-3.58	-3.52
Dedd2	2.52	2.70	Brp17	-3.57	-5.15
Slc19a1	2.51	4.04	Clpx	-3.53	-3.41
Kng1	2.37	2.24	Lonp2	-3.46	-2.47
Cln5	2.26	1.66	Gstm6	-3.45	-2.49
Coq10b	2.24	5.73	MAp19	-3.38	-3.61
Ndg2	2.23	2.04	D730039F16Rik	-3.36	-1.89
Igfbp2	2.20	6.39	Cyb5r3	-3.23	-1.73
C77032	2.18	2.25	D9Wsu20e	-3.10	-2.98
Ilvbl	2.07	2.24	2010305C02Rik	-3.04	-5.77
Nhlrc2	2.02	3.28	Cklfsf6	-2.84	-2.38
Rsn	2.02	2.74	BC027342	-2.77	-5.08
Glrx	1.97	4.30	Hsd17b2	-2.67	-4.24
Dnajb6	1.96	2.99	Tmem19	-2.64	-2.84
Htatip2	1.94	3.63	Cd59b	-2.62	-2.13
Atp5b	1.85	1.92	Mug1	-2.47	-1.91
Slc25a28	1.80	2.72	Hes6	-2.46	-1.79
Cox10	1.73	2.82	AW491445	-2.46	-3.64

Vdac2	1.72	2.22	Tmie	-2.42	-4.00
Vti1a	1.70	2.29	Carhsp1	-2.41	-1.57
Naca	1.59	2.61	Oprs1	-2.29	-3.87
			Dncl2a	-2.28	-1.79
Stard4	2.66	-2.59	AU022252	-2.22	-2.24
2610039E05Rik	1.67	-2.83	Abcb11	-2.15	-3.59
			Psen2	-2.01	-6.69
Pde4dip	-1.92	1.56	C9	-1.99	-5.13
Hsd3b2	-4.94	2.45	Hsd3b7	-1.89	-6.11
			Copz1	-1.68	-2.27

Table S5c, related to Figure 3m. List of significant genes shared in the livers of EOW, 2WM, and CR40 mice

	EOW-SD	2WM-SD	CR40-AL
**Symbol**	**zratio**	**zratio**	**zratio**
Pck1	7.70	9.07	5.06
Zfp259	4.23	2.27	2.89
Rpl3	3.57	2.44	1.55
3010033P07Rik	3.37	2.48	2.92
Idh2	3.27	3.66	3.65
Aldoc	2.93	4.65	2.43
Capn10	1.85	2.68	2.76
Slc25a5	1.66	1.98	2.30
Pla2g12a	2.24	-3.16	4.38
Fdps	4.92	10.26	-1.63
Hmgcs1	4.61	9.09	-4.78
Mvd	4.12	9.31	-2.74
Plac8	3.28	6.57	-2.76
Prelp	2.13	3.66	-3.63
Bbc3	1.93	5.79	-2.30
Foxo6b	1.62	2.33	-2.32
Tgoln1	-3.56	-3.80	1.56
Dhrs8	-3.16	-3.08	2.44
Akr1c19	-2.29	-3.21	4.17
Prox1	-1.86	-3.22	2.82
Bet1	-2.07	2.25	-1.82
1300013D18Rik	-5.77	-4.65	-3.03
1600023A02Rik	-5.59	-4.77	-6.85
Acbd4	-2.83	-2.51	-1.58
Acy3	-3.09	-4.08	-2.18
Gck	-5.00	-5.34	-6.75
Klkb1	-3.06	-2.26	-1.84
Rtp3	-5.62	-3.68	-2.13
Saa4	-4.25	-5.26	-5.46
Slc17a3	-3.08	-2.68	-3.43
Ube2n	-2.59	-1.89	-1.68

The zratios of significantly up- (red font) and down- (blue font) regulated genes in each pairwise comparison are shown. Significance is defined as zratio > 1.5 in either direction, false discovery rate (fdr) < 0.3 and p < 0.05. N = 4-6 mice per experimental group. EOW, metformin treatment every-other-week; 2WM, metformin treatment for two consecutive weeks each month; SD, standard diet; CR40, 40% calorie restriction; AL, *ad libitum* standard diet.

Table S6A, related to Figure 4b. List of significant liver metabolites in the EOW-SD and 2WM-SD pairwise comparisons

LIVER	Fold change ± s.e.m.	
**BinBase name**	**EOW-SD**	**2WM-SD**
N-carbamoylaspartate	4.75 ± 1.82	5.97 ± 1.68
5-aminovaleric acid	4.65 ± 1.62	3.60 ± 0.72
3-phosphoglycerate	2.14 ± 0.20	1.78 ± 0.12
beta-alanine	2.02 ± 0.52	1.52 ± 0.18
2-monooelin	0.33 ± 0.07	0.54 ± 0.13
aspartic acid	1.88 ± 0.46	
saccharic acid	1.38 ± 0.10	
lactamide	0.59 ± 0.07	
lactic acid	0.52 ± 0.06	
dihydrocholesterol	0.52 ± 0.15	
palmitoleic acid	0.40 ± 0.10	
cysteine-glycine	0.28 ± 0.07	
sucrose		2.59 ± 0.52
UDP-GlcNAc		2.23 ± 0.34
orotic acid		2.22 ± 0.47
pyrophosphate		2.01 ± 0.17
beta-sitosterol		1.91 ± 0.17
succinic acid		1.78 ± 0.38
ribulose-5-phosphate		1.72 ± 0.26
glycolic acid		1.68 ± 0.19
benzoic acid		1.45 ± 0.18
arachidic acid		1.40 ± 0.13
alanine		1.30 ± 0.14
stearic acid		1.25 ± 0.11
valine		0.76 ± 0.05
leucine		0.73 ± 0.06
pantothenic acid		0.72 ± 0.09
asparagine		0.68 ± 0.08
xanthosine		0.52 ± 0.05
ornithine		0.50 ± 0.08
shikimic acid		0.49 ± 0.12
galactose-6-phosphate		0.44 ± 0.06
tartaric acid		0.27 ± 0.04

Table S6b, related to Figure 4b. List of significant serum metabolites in the EOW-SD and 2WM-SD pairwise comparisons

SERUM	Fold change ± s.e.m.	
**BinBase name**	**EOW-SD**	**2WM-SD**
indole-3-lactate	4.34 ± 1.47	2.47 ± 0.67
3-hydroxybutyric acid	3.68 ± 0.86	2.19 ± 0.34
2,3-dihydroxybutanoic acid NIST	3.64 ± 0.72	1.90 ± 0.38

2-hydroxy-2-methylbutanoic acid	3.09 ± 0.82	1.74 ± 0.22
malic acid	2.93 ± 0.24	2.66 ± 0.28
succinic acid	2.91 ± 0.36	3.43 ± 0.51
fumaric acid	2.82 ± 0.24	2.51 ± 0.30
2-hydroxyhexanoic acid	2.09 ± 0.19	1.57 ± 0.23
lactamide	1.96 ± 0.25	1.97 ± 0.19
2-hydroxyglutaric acid	1.89 ± 0.28	2.30 ± 0.27
alanine	1.56 ± 0.24	1.47 ± 0.13
tyrosine	0.57 ± 0.03	0.67 ± 0.04
ornithine	0.48 ± 0.07	0.46 ± 0.07
allantoic acid	6.60 ± 2.44	
indoxyl sulfate	5.48 ± 2.20	
saccharic acid	2.95 ± 0.93	
deoxycholic acid	2.47 ± 0.43	
erythritol	2.40 ± 0.50	
uridine	2.26 ± 0.35	
cystine	1.89 ± 0.28	
hexuronic acid	1.83 ± 0.38	
isocitric acid	1.72 ± 0.24	
2-hydroxybutanoic acid	1.69 ± 0.30	
citric acid	1.64 ± 0.26	
glutamine	1.41 ± 0.17	
tryptophan	0.64 ± 0.06	
diglycerol	0.62 ± 0.10	
glycerol-alpha-phosphate	0.56 ± 0.10	
hexose	0.55 ± 0.16	
p-tosyl glucuronide	0.27 ± 0.04	
inosine		9.92 ± 4.38
beta-sitosterol		2.12 ± 0.30
tocopherol alpha		1.63 ± 0.14
benzoic acid		0.76 ± 0.05
methionine sulfoxide		0.72 ± 0.06
phenylalanine		0.68 ± 0.05
glycolic acid		0.67 ± 0.03
thymine		0.45 ± 0.09
creatinine		0.42 ± 0.08
cholic acid		0.41 ± 0.07
tartaric acid		0.21 ± 0.04

The fold change of significantly up- (red font) and down- (blue font) regulated metabolites in each pairwise comparison is shown.

Supplementary Methods

Physical performance tests. Latency to fall from a rotarod, sole wire-hang and wire cage top and forelimb grip strength were measured in mice after 16 to 18 weeks on diet. Rotarod: mice were given a habituation trial where they were placed on a rotating rod (Med Associates, Inc., St. Albans, VT) at a constant speed of 4 r.p.m. and had to remain on it for 1 min before the accelerating rod trial. Time to fall from a rod that accelerates from 4 to 40 r.p.m. over a 5-min period was recorded and averaged over three trials on the same day. There was a 30-min rest period between each trial. Wire hang test: the animal was left hanged in a 3-mm thick wire for a maximum of 1 min. Three attempts were recorded the same day and times were averaged. There was a 30-min rest period between each trial. Wire cage top: The animal was placed on a wire lid of a conventional housing cage and the lid was then inverted. The latency to when the animal falls was recorded and three attempts on the same day were averaged. The maximum trial length was 1 min and there was a 30-min rest period between each trial. Grip strength: The forelimb grasping applied by the mouse on a grid that is connected to a sensor (Columbus Instruments, Columbus, OH) was recorded. Five trials were carried out rejecting the highest and lowest values and averaging the other three.

Tissue collection. A midline incision was made in the abdomen of anesthetized mice and the liver exposed. Blood was collected from the inferior vena cava and immediately after the liver was perfused in situ with Krebs–Henseleit buffer at low pressure to wash blood out of the sinusoids. Following this, liver, kidneys, white adipose tissue, brown adipose tissue and heart were excised and weighed. Liver portions were saved for biochemistry, histochemistry, scanning electron microscopy, microarray and metabolomics analyses.

Histology. At sacrifice, representative tissue sections from the liver and kidneys were fixed in 10% neutral buffered formalin (Electron Microscopy Sciences, Hatfield PA) and then embedded in paraffin. Sections of 10 μm were cut and mounted onto slides. Sections were stained with Hematoxylin and Eosin (H&E) and Periodic acid Schiff (PAS) stain according to standardized protocols. Quantification of hepatic steatosis in H&E sections and PAS staining was performed by three independent observers blinded to the treatment groups. This was performed on at least 10 fields per animal with the data representing the average of two scores. Steatosis was determined by assigning an equally weighted arbitrary score of 0 (no visible steatosis), 1 (< 33% of the section showing steatosis), 2 (>33% but < 66% of the section showing steatosis) to 3 (>66% of the section showing steatosis). PAS staining was determined by assigning an equally weighted arbitrary score of 0 (no visible PAS staining), 1 (< 33% of the section showing PAS staining), 2 (>33% but < 66% of the section showing PAS staining) to 3 (>66% of the section showing PAS staining).

Kidney sections from all groups of mice (SD, n=10; EOW, n=6; 2WM, n=6) were examined and scored for possible toxic lesions by a certified veterinarian pathologist. Both H&E and PAS stained slides were evaluated (see above). For tubulointerstitial lesions, the severity score was as followed: 1 -mild (patches of tubules with smaller basophilic renal epithelial cells (RTE) and no distortion of the renal capsule); 2-moderate (more obvious atrophy and attenuation of RTE. Basement membrane thickening. Foci take up about ½ of a cluster of proximal tubules. Mild distortion of capsular surface); 3- severe (patches of damaged tubules take up entire groups of proximal tubules. Most of the tubules in the patch have severe attenuation of RTE). In addition, the presence or absence of necrotic RTE, swollen RTE, basement membrane pigmentation, intraluminal casts, and inflammation were noted. For glomerular lesions, the severity score was as followed: 1 –mild (focal to multifocal segmental mesanglial matrix expansion); 2 –moderate (global mesanglial matrix expansion resulting in lobulation of glomerular tuft); 3 –severe (synechia, crescent formation and obsolescence). The presence of hyaline material in glomerular

tufts (hyaline thrombi) was also noted. With regard to the distribution of lesions, a score 0 indicated no lesion; 1, up to 1/3 of tubules or glomeruli affected; 2, 1/3 – 2/3 affected; and 3, >2/3 affected.

Electron microscopy. Livers were perfused with erythrocyte-free oxygenated Krebs-Henseleit bicarbonate buffer (95% O_2–5% CO_2, 37°C) until clear of blood, then a portion of the liver was subjected to needle perfusion (26G 1mL syringe) with fixative (2% glutaraldehyde–3% paraformaldehyde in 0.1 M sodium cacodylate buffer (0.1 M sucrose, 2 mM $CaCl_2$). Three to five randomly selected blocks from each animal were then dried and stained, mounted onto stubs, and sputter coated with platinum, as described previously[75]. A Jeol 6380 Scanning Electron Microscope (JEOL Ltd, Tokyo, Japan) at a resolution of 15,000× was used to examine fenestrations in the liver sinusoidal endothelium. Endothelial porosity is the percentage of the endothelial surface perforated by fenestrations and is calculated from their diameter and frequency.

Microarray method. Total RNA quantity and quality was tested using the Agilent Bioanalyzer RNA 6000 Chip (Agilent, Santa Clara, CA). Five hundred ng total RNA was labeled according to the manufacturer's instructions using the Illumina® TotalPrepTM RNA amplification kit (Illumina, San Diego, CA). A total of 750 ng biotinylated aRNA was hybridized to the Illumina Mouse Ref-8 v2 BeadChip overnight. Following posthybridization rinses, arrays were incubated with streptavidin-conjugated Cy3, and scanned at a resolution of 0.53 µm using an Illumina iScan scanner. Hybridization intensity data were extracted from the scanned images using Illumina BeadStudio GenomeStudio software, V2011.1. Raw data were subjected to Z-normalization, as described elsewhere[73,76]. Principal component analysis (PCA) was performed on the normalized Z-scores of all of the detectable probes in the samples using DIANE 6.0 software, available from: (http://www.grc.nia.nih.gov/branches/rrb/dna/ diane_software.pdf). Significant genes were selected by the z-test < 0.05, false discovery rate < 0.30, as well as z-ratio > 1.5 in both directions and ANOVA p value < 0.05.

For the calculation of pairwise distances between samples, each microarray was considered as a point in a high-dimensional space since we treated each probe as a variable. For parametric analysis of gene set enrichment (PAGE), our expression data was tested using the PAGE method as previously described[77]. Briefly, for each pathway under each pair of conditions, an aggregated Z score was computed as:

$$= \frac{\frac{Z\text{-}score\ pathway}{n_{\text{pathway}}} Z\text{-}ratio\ genes\ in\ the\ pathway - Z\text{-}ratio\ genes\ on\ the\ array}{\sigma_{\text{sample}}}$$

Where n_{pathway} is the number of genes in the specific pathway and σ_{sample} is the standard derivation of Z-ratio on the comparison sample arrays. For each Z (pathway) a P value was computed (JMP 6.0 software) to the total Z-ratio in comparison by Z-test. Ingenuity Pathways Analysis© was performed by using the tools supplied by Ingenuity Inc. (Ingenuity Systems; Redwood City, CA).

Liver microarray data from our recent study[32] on the effect of chronic 40% calorie restriction in 2-year-old male C57BL/6J mice versus *ad libitum* (AL) feeding (accession number GEO: GSE81959) was extracted and used to show the number of genes that overlapped with the intermittent metformin treatment (EOW-SD; 2WM-SD). Both control groups were comparable, as these mice (AL and SD) were of the same strain and age, and fed standard diet ad libitum. In the two studies, the Illumina Mouse Ref-8 v2 BeadChip was used, and Z-normalization of the raw data was carried out, enabling the selection of significant genes by

the use of z-test, false discovery rate, z-ratio, and ANOVA. This exploratory data analysis was aimed only at visualizing trends in gene expression, using Venn diagrams, heatmap representation, and pie charts.

Quantitative RT-PCR. Total RNA was extracted from frozen tissue with Trizol® Reagent (Thermo Fisher Scientific, Waltham, MA). Briefly, 50-100mg of liver was homogenized in 1 mL Trizol® Reagent and phase separation was carried out with 0.2mL of chloroform. Aqueous phase is collected and RNA is precipitated with 100% isopropanol. Pellet is washed twice with 75% ethanol and let it dry. RNAis resuspended in RNAse free water and extracted quantity is measured with a NanoDrop 1000 spectrophotometer (Thermo Fisher Scientific, Waltham, MA). 1µg of RNA was reverse-transcribed using iScript™ cDNA Synthesis Kit (Bio-Rad Laboratories, Inc., Hercules, CA) according to the manufacturer's instructions. iTaq™ Universal SYBR® Green Supermix (Bio-Rad Laboratories, Inc., Hercules, CA) was used for quantitative PCR reactions, and samples were analyzed on the StepOnePlus™ Real-Time PCR System (Applied Biosystems). Oligonucleotide sequences were as follows: LPIN1 (NM_172950.2), 5'- CCTTCTATGCTGCTTTTGGGAACC-3' and 5'- GTGATCGACCACTTCGCAGAGC-3'; MUP5 (NM_008649.2), 5'- CAGCTGATGGAGCTCTTTGGT-3' and 5'- GCGATTGGCATTGGATAGGT-3'; SQLE (NM_009270.2), 5'- GGAGGCTACCGTGTTCTCCA-3' and 5'- CTGCACTTGGTTGGTTTCTGAC-3'; GOT1 (NM_010324.1), 5'- AACGACAACAGCCTCAACCA-3' and 5'- ATGGTTCTCCCAGGTTGGTG-3'; NSDHL (NM_010941.3), 5'- AGCAGTGCCAGTGTTGTCTT-3' and 5'- GCATCCAGTACTGCTCTCTCC-3'; GPD2 (NM_010274.2), 5'-ACAATAGCAGGTGGGAAGTGG-3' and 5'- CCAAGTTCTCCTCGGCAGTT-3'.

Western blotting. Frozen liver tissues were lysed in radioimmunoprecipitation buffer containing EGTA and EDTA (Boston BioProducts, Ashland, MA, USA) supplemented with protease inhibitor cocktail (Sigma-Aldrich) and phosphatase inhibitor cocktail sets I and II (Calbiochem, San Diego, CA, USA). Protein concentration in clarified lysates was determined using the bicinchoninic acid reagent (Pierce BCA Protein Assay Kit, Thermo Fisher Scientific, Waltham, MA, USA). Proteins (20 µg/well) were separated on 4-15% Criterion TGX precast gels (BioRad, Hercules, CA, USA) using SDS-polyacrylamide gel electrophoresis under reducing conditions and then electrophoretically transferred onto nitrocellulose membranes (Trans-Blot Turbo Transfer System, BioRad). Western blots were performed according to standard methods, which involved a blocking step in phosphate-buffered saline/0.1% Tween-20 (PBS-T) supplemented with 5% non-fat milk and incubation with primary antibodies of interest. All antibodies were detected with horseradish peroxidase-conjugated secondary antibodies (Santa Cruz Biotechnology, Dallas, TX, USA) and visualized by enhanced chemiluminescence (Immobilon Western Chemiluminescent HRP Substrate, Millipore, Billerica, MA, USA). Imaging of the signal was captured with Amersham Imager 600 (GE Healthcare, Piscataway, NJ, USA). Quantification of the protein bands was performed by volume densitometry using ImageJ software (National Institutes of Health, Bethesda, MD, USA). The primary antibodies used in this study were raised against AMPK (cat.: 2532S, Cell Signaling Technology, Danvers, MA, USA), phosphoThr-172 AMPK (cat.: 2531S, Cell Signaling), ACC (cat.: 04-322, Millipore), and phosphoSer-79 ACC (cat.: 07-303, Millipore). The antibodies were used at the dilution (1:1000) recommended by the manufacturers.

Metabolomics. Liver tissue (4mg) was homogenized in extraction solution by mixing acetonitrile, isopropanol and water in proportions 3:3:2 (JT Baker, Center Valley PA), then vortexed for 45 s and then 5 min at 4C. Following centrifugation for 2 min at 14,000 rcf, two aliquots of the supernatant (500µL each aliquot) were made for analysis and one for backup. One aliquot was dried via evaporation overnight in the Labconco Centrivap cold trap concentrator (Labconco, Kansas City MO). The dried aliquot was then re-

suspended with 500µL 50% acetonitrile (degassed as given), then centrifuged for 2 min at 14,000 rcf using the centrifuge Eppendorf 5415. The supernatant was moved to a new Eppendorf tube and again evaporated to dryness. Internal standards (C08-C30, fatty acid methyl esters) were then added and the sample was derivatized by methoxyamine hydrochloride in pyridine and subsequently by N-methyl-N-trimethylsilyltrifluoroacetamide for trimethylsilylation of acidic protons. Data were acquired using the method as described in (Fiehn et al., 2008). Briefly, metabolites were measured using a Restek corporation rtx5Sil-MS column (Restek Corporation; Bellefonte PA; 30 m length x 0.25 mm internal diameter with 0.25µm film made of 95% dimethly/5%diphenylpolysiloxane) protected by a 10m long empty guard column which is cut by 20cm intervals whenever the reference mixture QC samples indicate problems caused by column contaminations. This sequence of column cuts has been validated by UC Davis Metabolomics Core with no detrimental effects detected with respect to peak shapes, absolute or relative metabolite retention times or reproducibility of quantifications. This chromatography method yields excellent retention and separation of primary metabolite classes (amino acids, hydroxyl acids, carbohydrates, sugar acids, sterols, aromatics, nucleosides, amines and miscellaneous compounds) with arrow peak widths of 2–3s and very good within-series retention time reproducibility of better than 0.2s absolute deviation of retention times. The mobile phase consisted of helium, with a flow rate of 1 mL/min, and injection volume of 0.5µL. The following mass spectrometry parameters were used: a Leco Pegasus IV mass spectrometer with unit mass resolution at 17 spectra s-1 from 80-500 Da at -70 eV for elution of metabolites. As a quality control, for each sequence of sample extractions, one blank negative control was performed by applying the total procedure (i.e. all materials and plastic ware) without biological sample. Result files were transformed by calculating the sum intensities of all structurally identified compounds for each sample (i.e. those signals that had been positively identified in the data pre-processing schema outlined above), and subsequently dividing all data associated with a sample by the corresponding metabolite sum. The resulting data were multiplied by a constant factor in order to obtain values without decimal places. Intensities of identified metabolites with more than one peak (e.g. for the syn- and anti-forms of methoximated reducing sugars) were summed to only one value in the transformed data set. The original non-transformed data set was retained. The general concept of this data transformation is to normalize data to the 'total metabolite content', but disregarding unknowns that might potentially comprise artifact peaks or chemical contaminants.

Sample preparation for analysis and quantification of metformin. 1) Mouse serum analysis. To 20 µl serum, 5 µl internal standard (IS) solution (3200 ng/ml phenformin (Sigma-Adrich) in methanol), 5 µl calibration standards (40-20000 ng/ml), QCs, and study samples. For blanks, 5 µl of methanol were added to the blank mouse serum in place of the IS solution. 2) Mouse liver samples. Liver samples were stored frozen at -70°C and thawed to room temperature, then homogenized in phosphate-buffered saline (PBS) using a probe sonicator (30 s at 50% maximal amplitude), using 4 ml PBS per gram liver tissue (ml/g). To 100 µl liver homogenates, 20 µl IS solution, 20 µl ammonium hydroxide solution (15% in water), and 400 µl acetonitrile were added to the standards, QCs, and study samples. For blanks, 20 µl of methanol were added to the blank mouse serum in place of the IS solution. For both serum and liver samples, these mixtures were vortexed for 10 min on a multi-tube vortex mixer at maximal speed, and the suspensions were then clarified by centrifugation (18000 g, 10 min). Five (serum) and eight (liver) microliters of the resulting supernatants were added to 1000 µl of 90% methanol in water, and then these mixtures were transferred to HPLC vials for LC-MS/MS analysis as described below. Accuracy and precision during sample analysis for metformin in mouse serum and liver homogenates were as followed: serum (94.0% accuracy, and CV of 10.1%) and liver homogenate (100% accuracy, and CV of 9.17%).

Chromatographic analysis. The mixtures were analyzed using a system composed of a Shimadzu LC-20AD HPLC pumps equipped with a CTC-PAL autosampler set at 5 °C, and API Sciex 5500 mass spectrometer supplied with electrospray ionization (ESI). Multiple reaction monitoring (MRM) chromatograms were acquired and quantitated using AB Sciex Analyst Software, version 1.6.2. For the LC conditions, a Phenomenex Kinetex HILIC column (50 x 2.1 mm, 2.6 µm) was used and operated at 40 °C. Gradient elution was used for the separation, with solvent (A) composed of 5 mM ammonium formate in water and solvent (B) 5 mM ammonium formate in methanol. The solvent gradient in volumetric ratios of solvents A and B was as followed: 0–2 min, 100 A/0 B; 2–4 min, 40 A/60 B; 4-6 min, 0 A/100 B. The flow rate was 0.2 $mL \cdot min^{-1}$ and the injection volume was 10 µL. The analytes were monitored in the positive-ion mode for MRM transitions at m/z 130 →71.0 (metformin) and m/z 206.2 →105.0 (cyproheptadine, used as internal standard).
